# The Prevalence of Contact Allergy to Formaldehyde and Formaldehyde Releasers: A Systematic Review and Meta‐Analysis

**DOI:** 10.1111/cod.70172

**Published:** 2026-04-26

**Authors:** Kian Karimian, Daniel Isufi, Mikkel Bak Jensen, Rebekka Søgaard, Jeanne Duus Johansen, Jakob Ferløv Baselius Schwensen

**Affiliations:** ^1^ National Allergy Research Centre, Department of Dermatology and Allergy, Herlev and Gentofte Hospital Hellerup Denmark; ^2^ Department of Dermatology and Allergy Herlev and Gentofte ‐ Copenhagen University Hospital Copenhagen Denmark; ^3^ Institute of Clinical Medicine, Faculty of Health and Medical Sciences University of Copenhagen Copenhagen Denmark

**Keywords:** allergic contact dermatitis, contact allergy, dermatitis, formaldehyde, formaldehyde releasers, preservative

## Abstract

Formaldehyde and its releasers are common preservatives and potent sensitizers. This meta‐analysis aimed to estimate the prevalence of formaldehyde contact allergy and allergy to its five most common releasers among dermatitis patients. Two authors independently searched PubMed, Embase, and Web of Science from inception to 30th September 2025. A proportion meta‐analysis was conducted using a random‐effects model. A total of 158 studies involving 1 347 638 dermatitis patients were included. The pooled prevalence of formaldehyde contact allergy was 2.88% (95% CI 2.55–3.24) with clinical relevance at 41.57%. The pooled prevalence for releasers was 2.76% for 2‐bromo‐2‐nitropropane‐1,3‐diol, 1.89% for quaternium‐15, 1.42% for diazolidinyl urea, 1.37% for DMDM hydantoin, and 1.20% for imidazolidinyl urea. Quaternium‐15 showed the highest clinical relevance at 55.67%. Significant sensitization rates were observed in different geographic regions, with the highest prevalence found in North America (6.8%). The prevalence rate was 2.96% among children and 2.61% among adults, with no significant difference. The prevalence rate was 2.65% among patients with atopic dermatitis (AD) and 2.85% among patients without AD, with no significant difference. The profound geographic disparities suggest that regulatory interventions, such as the EU's prohibition of formaldehyde and quaternium‐15 in cosmetics, are highly effective public health measures.

**Abbreviations**
AXIS: Appraisal tool for cross‐sectional studies
DMDM hydantoin: 1,3‐dimethylol‐5,5‐dimethylhydantoin
MCI/MI: Methylchloroisothiazolinone/methylisothiazolinone
MI: Methylisothiazolinone
PPT: Positive Patch Test
PRISMA: Preferred Reporting Items for Systematic Reviews and Meta‐analyses
PROSPERO: International Prospective Register of Systematic Reviews

## 1 Introduction

Formaldehyde is a widespread preservative and potent sensitizer that remains a significant public health challenge. Formaldehyde and formaldehyde releasers are a class of related chemicals widely used as cost‐effective preservatives to prevent microbial growth in a vast range of consumer and industrial products, including cosmetics, personal care items, and textiles [1, 2]. Formaldehyde is recognized as an indoor air pollutant due to its toxicological profile [3].

Contact allergy to formaldehyde is a well‐documented and persistent problem among dermatitis patients. The prevalence of sensitization in populations that have undergone patch testing ranges from 2% to 3% [4, 5, 6, 7] in Europe to 8% in the United States (US) [8, 9].

Since 2019, formaldehyde and quaternium‐15 have been prohibited as direct cosmetic ingredients in the European Union (EU) [10]. In contrast, these preservatives are still permitted in the US. However, the use of other formaldehyde‐releasing preservatives, including 1,3‐dimethylol‐5,5‐dimethylhydantoin (DMDM hydantoin) and diazolidinyl urea, has not been prohibited. These preservatives are permitted under the EU restriction (Annex V) [11], and a “releases formaldehyde” warning label is required only if the free formaldehyde concentration in the final product exceeds 10 ppm [12]. While this is progress from the previous limit of 500 ppm, the distinction in regulation imposes a substantial burden on sensitized individuals, who must learn to recognize a long list of chemically diverse formaldehyde releasers rather than a single allergen.

While studies have documented regional prevalence rates, a large‐scale analysis directly comparing sensitization patterns to this entire class of allergens in the context of these differing regulatory environments is lacking. This study aims to provide a comprehensive epidemiological understanding of contact allergy to formaldehyde and its five common releasers in patients with dermatitis.

## 2 Methods

Prior to initiation, a study protocol was registered on the Prospective Register of Systematic Reviews (PROSPERO; CRD420251151165). The study adhered to the Preferred Reporting Items for Systematic Reviews and Meta‐Analyses (PRISMA) guidelines [13].

### 2.1 Literature Search

Three databases (PubMed, Embase, and Web of Science) were searched from inception through 30th September 2025 using the search strategy available in Supplementary Table 1. All titles and abstracts were extracted and imported into the web‐based screening tool Rayyan [14], and duplicates were manually removed.

### 2.2 Inclusion and Exclusion Criteria

Two authors (K.K. and D.I.) independently assessed studies for eligibility. Any disagreements were resolved through discussion with the senior author (J.F.B.S.).

Inclusion criteria were: (I) Original studies, (II) written in any language, (III) reporting the number of dermatitis patients undergoing consecutive patch testing, and (IV) reporting the number of positive patch tests (PPTs) to formaldehyde and formaldehyde releasers.

Exclusion criteria were: (I) grey literature (i.e., letters and conference abstracts) and (II) studies not conducted on dermatitis patients (i.e., general population). If multiple publications involving the same population were retrieved, the most comprehensive report was selected for inclusion. Studies for which eligibility could not be determined from the title or abstract alone were retained for full‐text assessment.

### 2.3 Data Extraction and Quality Assessment

Data were extracted independently by K.K. and D.I. into pre‐defined tables including information on the first author's surname, publication year, study period, country, concentration, vehicle, number of PPTs, sex distribution (male, %), age (mean, standard deviation [SD]), patients with AD (%), and clinically relevant PPTs (%). The study quality of cross‐sectional studies was assessed using the Appraisal tool for Cross‐Sectional Studies (AXIS) [15].

### 2.4 Statistical Analysis

Statistical analyses were performed using RStudio (version 2025.05.0, build 496). Pooled prevalence estimates and 95% confidence intervals (CIs) were calculated using a random‐effects model with inverse variance weighting. The DerSimonian–Laird estimator was used to estimate between‐study heterogeneity for tau^2^, and the Jackson method was used to calculate CIs for tau^2^ and tau. The *I* ^2^ statistic, which estimates the percentage of total variation attributable to heterogeneity rather than chance, was derived from the Q statistic. All proportions were logit‐transformed prior to meta‐analysis to stabilize the variance and minimize the influence of extreme proportions. For individual studies, 95% CIs were calculated using the Clopper–Pearson exact method.

To prevent duplicate patient populations from being included, studies from the same center with overlapping study periods were assessed. In cases where data overlap was suspected, only the study reporting the largest sample size was included. Some studies reported on distinct patient cohorts tested with different concentrations. If a study provided separable data for these cohorts, the data were extracted as distinct entries. Consequently, the same author and year may appear multiple times in the analyses. If data for different concentrations could not be disaggregated, the result was included in the overall pooled analysis but excluded from the concentration‐specific sub‐analysis. For the sub‐analysis of concentrations, a specific test concentration was only included if it was reported in at least five studies. Studies that reported data from more than one region were excluded from the regional sub‐analysis.

## 3 Results

### 3.1 Qualitative Assessment of the Included Studies

#### 3.1.1 Eligible Studies

**FIGURE 1:**
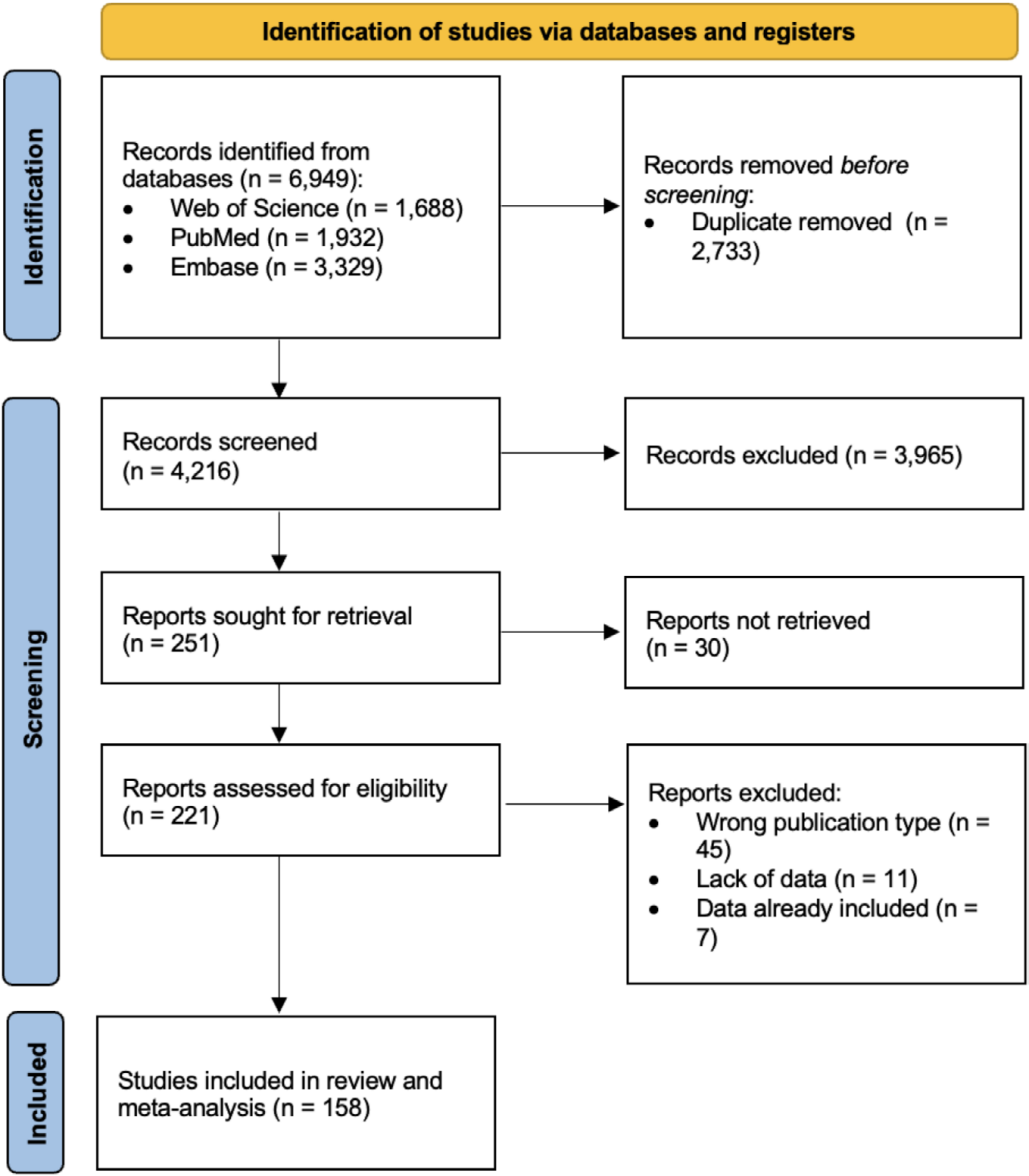
The Preferred Reporting Items for Systematic Reviews and Meta‐analyses (PRISMA) flowchart.

In total, 6949 articles (PubMed = 1932, Embase = 3329, and Web of Science = 1688) were retrieved. After removing duplicates, a total of 4216 non‐duplicate articles were screened based on the title and abstract. Of these, 221 were included for full‐text assessment. Based on the full‐text articles, 63 were excluded for various reasons, yielding 158 articles included in the meta‐analysis (Figure 1).

#### 3.1.2 Characteristics of the Included Studies

A total of 1 347 638 patients were included (*n* = 158 studies [4, 5, 6, 7, 8, 16, 17, 18, 19, 20, 21, 22, 23, 24, 25, 26, 27, 28, 29, 30, 31, 32, 33, 34, 35, 36, 37, 38, 39, 40, 41, 42, 43, 44, 45, 46, 47, 48, 49, 50, 51, 52, 53, 54, 55, 56, 57, 58, 59, 60, 61, 62, 63, 64, 65, 66, 67, 68, 69, 70, 71, 72, 73, 74, 75, 76, 77, 78, 79, 80, 81, 82, 83, 84, 85, 86, 87, 88, 89, 90, 91, 92, 93, 94, 95, 96, 97, 98, 99, 100, 101, 102, 103, 104, 105, 106, 107, 108, 109, 110, 111, 112, 113, 114, 115, 116, 117, 118, 119, 120, 121, 122, 123, 124, 125, 126, 127, 128, 129, 130, 131, 132, 133, 134, 135, 136, 137, 138, 139, 140, 141, 142, 143, 144, 145, 146, 147, 148, 149, 150, 151, 152, 153, 154, 155, 156, 157, 158, 159, 160, 161, 162, 163, 164, 165, 166, 167, 168]). Of these, 329 591 (24.46%) were male. The mean age across the studies was 38.34 ± 13.55 years (*n* = 49 studies [8, 16, 17, 18, 19, 20, 21, 22, 23, 24, 25, 26, 27, 28, 29, 30, 31, 32, 33, 34, 35, 36, 37, 38, 39, 40, 41, 42, 43, 44, 45, 46, 47, 48, 49, 50, 51, 52, 53, 54, 55, 56, 57, 58, 59, 60, 61, 62, 63]). The number of patients with AD was 127 446 (9.46%) (*n* = 70 studies [6, 16, 19, 20, 23, 24, 26, 28, 29, 31, 32, 34, 38, 42, 43, 47, 48, 50, 53, 54, 56, 57, 58, 60, 61, 63, 64, 65, 66, 67, 68, 69, 70, 71, 72, 73, 74, 75, 76, 77, 78, 79, 80, 81, 82, 83, 84, 85, 86, 87, 88, 89, 90, 91, 92, 93, 94, 95, 96, 97, 98, 99, 100, 101, 102, 103, 104, 105, 106, 107]). The studies were conducted in Europe (*n* = 70 studies [4, 5, 6, 7, 18, 28, 32, 37, 39, 43, 48, 54, 58, 61, 63, 67, 70, 71, 72, 73, 77, 78, 80, 82, 83, 84, 87, 88, 89, 91, 92, 94, 96, 98, 101, 105, 107, 108, 109, 110, 111, 112, 113, 114, 115, 116, 117, 118, 119, 120, 121, 122, 123, 124, 125, 126, 127, 128, 129, 130, 131, 132, 133, 134, 135, 136, 137, 138, 139, 140]), Middle East (*n* = 16 studies [20, 22, 23, 35, 46, 55, 56, 60, 69, 100, 141, 142, 143, 144, 145, 146]), North America (*n* = 33 studies [8, 16, 17, 21, 24, 25, 27, 34, 36, 38, 41, 42, 50, 53, 64, 65, 66, 68, 75, 76, 79, 81, 85, 86, 93, 102, 147, 148, 149, 150, 151, 152, 153]), South America (*n* = 3 studies [51, 154, 155]), East Asia (*n* = 14 studies [19, 29, 31, 33, 40, 45, 52, 74, 95, 104, 156, 157, 158, 159]), Southeast Asia (*n* = 11 studies [30, 44, 49, 57, 59, 103, 106, 160, 161, 162, 163]), Oceania (*n* = 4 studies [90, 97, 99, 164]), Africa (*n* = 4 studies [26, 47, 62, 165]) (Table 1). Based on the AXIS assessment, most studies were judged to have a low risk of bias (Supplementary Table 2).

**TABLE 1:** Characteristics of the included studies.

First author surname [year]	Study period [year]	Country	Conc. [%] and vehicle^a^	Patients [n]	Male [n]	Male [%]	Age [median]	Age [mean ± SD]	Atopic dermatitis [n]	Atopic dermatitis [%]	Atopic dermatitis positive [n]
Aalto‐Korte [2021] [135]	2007–2020	Finland	1/2	1497	NA	NA	NA	NA	NA	NA	NA
Adams [1985] [64]	1977–1983	United States	NA	713	152	21	NA	NA	115	16	NA
Aguilar‐Bernier [2012] [84]	2005–2010	Spain	1	839	308	36.8	45	NA	133	15.9	NA
Akasya‐Hillenbrand [2002] [69]	1996–1999	Turkey	1	542	239	44.1	33.5	NA	15	2.8	NA
Albert [1998] [150]	1990–1997	United States	1	608	183	30.1	NA	NA	NA	NA	NA
Andernord [2022] [137]	2010–2017	Sweden	1/2	21 663	6700	31	44	NA	NA	NA	NA
Anderson [2007] [151]	2004–2005	United States	1	210	NA	NA	NA	NA	NA	NA	NA
Atwater [2021] [8]	1994–2016	Canada and United States	1&2	50 799	17 119	33.7	NA	48.0 ± 16.9	NA	NA	NA
Bauer [2023] [105]	2011–2020	11 European countries	1/2^b^	62 674	20 735	33.1	NA	NA	18 515	29.5	NA
Belhadj [2024] [62]	2009–2018	Tunisia	NA	832	393	47.2	38.93	38.9 ± 12.5	NA	NA	NA
Beliauskiene [2011] [78]	2006–2008	Lithuania	1	816	215	26.3	46	NA	144	17.9	67
Bilcha [2010] [26]	2007–2008	Ethiopia	NA	514	171	33.3	36.3	36.3 ± 12.1	8	1.5	NA
Bizjak [2022] [138]	2019–2021	Slovenia	2	748	198	26.5	45	NA	NA	NA	NA
Boonchai [2021] [103]	2010–2019	Thailand	2	792	261	33	NA	NA	88	11.1	NA
Boonchai [2011] [30]	1999–2008	Thailand	1	1247	239	19.2	38.5	38.5 ± 13.9	NA	NA	NA
Boonchai [2023] [59]	2000–2020	Thailand	1/2	5276	1137	21.6	NA	42.1 ± 15.2	NA	NA	NA
Bordel‐Gómez [2010] [28]	2000–2005	Spain	1	1092	419	38.4	41	41.4 ± 16.5	164	15	96
Boyvat [2005] [22]	2000–2004	Turkey	1	308	111	36.1	37.03	37.0 ± 14.5	NA	NA	NA
Boyvat [2021] [56]	2013–2019	Turkey	2	1309	545	41.6	41	40.9 ± 16.8	66	5	20
Brasch [2003] [70]	1995–2001	3 European countries	1	16 252	5514	33.9	NA	NA	2322	14.3	NA
Britton [2003] [71]	2000–2000	United Kingdom	1	3062	1043	34.1	NA	NA	1222	39.9	NA
Bruynzeel [2005] [116]	1996–2000	8 European countries	1	26 210	9077	34.6	NA	NA	NA	NA	NA
Carvalho [2024] [107]	2005–2021	Portugal	2	145	46	32.1	13	NA	63	43.4	31
Cheng [2011] [29]	2001–2006	China	1	1354	363	26.8	38	38.0 ± 14.1	63	4.6	NA
Cheng [2024] [163]	2012–2021	Singapore	1	4585	1738	37.9	42	NA	NA	NA	NA
Cheng [2014] [162]	2006–2011	Singapore	1	3177	1393	44	38	NA	NA	NA	NA
Chow [2013] [164]	1993–2006	Australia	1	6845	NA	NA	NA	NA	NA	NA	NA
Clemmensen [2014] [89]	2009–2013	Denmark	NA	2221	788	35.5	NA	NA	293	13.2	120
Collis [2020] [53]	2007–2018	United States	NA	29	NA	NA	NA	10.9 ± 5.1	18	62.1	NA
Dear [2021] [99]	2012–2018	Australia	NA	964	257	26.7	44.5	NA	240	24.9	NA
Dinkloh [2015] [91]	2006–2011	3 European countries	1	10 124	3063	30.3	NA	NA	2543	25.1	NA
Duarte [2013] [155]	2006–2011	Brazil	NA	618	195	31.6	NA	NA	NA	NA	NA
Dugonik [2021] [54]	2008–2017	Slovenia	2	15 171	4670	30.8	NA	50.3	124	9.6	NA
El‐Rab [1995] [146]	NA	Saudi Arabia	1	240	109	45.4	NA	NA	NA	NA	NA
Erfan [2015] [143]	2011–2014	Turkey	NA	169	107	63.3	41.06	NA	NA	NA	NA
Erkoç [2025] [140]	2018–2025	Turkey	2	62	28	45.2	69.5	NA	NA	NA	NA
Fasth [2018] [4]	2007–2016	Denmark	1&2	8463	NA	NA	NA	NA	NA	NA	NA
Felmingham [2020] [97]	1993–2017	Australia	NA	511	199	38.9	14	NA	212	41.5	NA
Fransway [2013] [86]	2007–2008	Canada and United States	1	5085	1812	35.6	NA	NA	1091	21.5	NA
Garg [2018] [44]	2013–2015	India	1^b^	58	8	13.8	36.28	36.3 ± 11.8	NA	NA	NA
Giménez‐Arnau [2017] [6]	2009–2012	12 European countries	1&2	57 146	19 201	33.6	NA	NA	10 952	22.5	NA
Hacioglu [2010] [141]	2007–2009	Turkey	1	73	7	9.6	37.5	NA	NA	NA	NA
Hasan [2005] [117]	1995–2002	Finland	1	NA	NA	NA	NA	NA	NA	NA	NA
Hauksson [2010] [122]	2006–2007	Sweden	0.03&0.10&0.32&1&2&3	1397	519	37.2	NA	NA	NA	NA	NA
Helsing [2010] [123]	2007–2008	Norway	NA	2089	NA	NA	NA	NA	NA	NA	NA
Herro [2011] [79]	NA	United States	NA	101	48	47.5	11.66	NA	54	53	48
Hervella‐Garcés [2016] [131]	2012–2012	Spain	1	3466	NA	NA	NA	NA	NA	NA	NA
Hirano [1982] [156]	1976–1979	Japan	2	256	NA	NA	NA	NA	NA	NA	NA
Hogan [1988] [148]	1983–1987	Canada	NA	542	212	39.1	NA	NA	NA	NA	NA
Hogeling [2008] [24]	1996–2006	Canada	1	100	38	38	13.7	13.7 ± 3.4	41	41	30
Holness [1995] [17]	1985–1989	Canada and United States	1	4055	1744	43	NA	43.8	NA	NA	NA
Huang [2019] [45]	1996–2015	Taiwan	1	757	213	28.1	NA	39.6	NA	NA	NA
Husain [1977] [108]	1970–1973	Scotland	2	1312	603	46	NA	NA	NA	NA	NA
Ibekwe [2019] [47]	2014–2018	Nigeria	2	81	31	38.3	26.5	26.5 ± 16.3	16	20	NA
Isaksson [2019] [168]	2014–2014	8 countries	1&2	2778	1062	38.2	NA	NA	NA	NA	NA
Isaksson [2014] [129]	2011–2011	Sweden	1&2	2122	698	32.9	NA	NA	NA	NA	NA
Isaksson [2015] [167]	2012–2012	12 countries	1	2259	776	NA	NA	NA	NA	NA	NA
Ito [2022] [104]	2015–2019	Japan	0.18^c^	5865	1334	22.7	NA	NA	497	8.5	NA
Jacob [2010] [27]	2004–2006	United States	0.5	45	21	46.7	8.21	8.2 ± 4.6	NA	NA	33
Jacobs [1995] [111]	1982–1993	United Kingdom	NA	21 265	8787	41.3	NA	NA	NA	NA	NA
Jong [2007] [120]	2004–2005	United Kingdom	1	6958	NA	NA	40	NA	NA	NA	NA
Kashani [2005] [20]	2002–2004	Iran	NA	250	60	24	32.7	32.7 ± 13.1	89	35.6	NA
Kasumagic‐Halilovic [2018] [134]	2016–2017	Bosnia‐Herzegovina	1	355	146	41.1	NA	NA	NA	NA	NA
Katran [2024] [145]	2018–2023	Turkey	NA	383	159	41.5	NA	NA	NA	NA	NA
Kieć‐Swierczyńska [2006] [118]	2000–2005	Poland	NA	1937	630	32.5	NA	NA	NA	NA	NA
Kieć‐Swierczyńska [2012] [127]	1996–2009	Poland	1	4433	1474	33.3	NA	NA	NA	NA	NA
Kieć‐Swierczyńska [1996] [112]	1990–1994	Poland	1	332	173	52.1	NA	NA	NA	NA	NA
Klimanska [2011] [125]	NA	Poland	1	50	25	50	NA	NA	NA	NA	NA
Koca [2021] [100]	2015–2017	Turkey	2	1169	487	41.7	39.1	NA	48	4.1	NA
Kręcisz [2015] [130]	2011–2013	Poland	1	405	97	24	NA	NA	NA	NA	NA
Kundak [2020] [144]	2013–2017	Turkey	NA	89	30	33.7	10.3	NA	NA	NA	NA
Lagrelius [2016] [132]	2011–2013	Sweden	NA	2285	1086	47.5	16.7	NA	NA	NA	NA
Lam [2008] [158]	1995–1999	Hong Kong	0.01	2585	1050	40.6	NA	NA	NA	NA	NA
Landeck [2011] [81]	1990–2006	United States	NA	1247	373	29.9	NA	NA	172	13.8	112
Latorre [2011] [82]	2005–2009	Spain	1	7838	2712	34.6	NA	NA	1105	14.1	NA
Lazarov [2006] [23]	1998–2004	Israel	1	2156	694	32	45.4	45.4 ± 17.1	235	10.9	NA
Li [2007] [74]	2003–2005	China	NA	599	180	30.1	NA	NA	14	2.3	NA
Lin [2021] [159]	1978–2018	Taiwan	NA	4005	1209	30.2	NA	NA	NA	NA	NA
Linauskienė [2017] [92]	2014–2015	Lithuania	1	297	40	13.5	NA	NA	70	23.6	44
Liu [1997] [157]	1988–1996	China	1	1135	312	27.5	NA	NA	NA	NA	NA
Lynde [1982] [147]	1972–1981	Canada	2	4190	NA	NA	NA	NA	NA	NA	NA
Machovcova [2012] [32]	2005–2006	Czech Republic	1	218	114	52.3	12.6	12.6	59	27	21
Machovcova [2005] [73]	1997–2001	Czech Republic	1	12 058	4416	36.6	41.7	NA	2062	17.1	NA
Mahfoudh [2017] [165]	2003–2010	Tunisia	1	1019	NA	NA	NA	NA	NA	NA	150
Malinauskiene [2015] [88]	2010–2012	Lithuania and Sweden	1	642	73	11.4	NA	NA	274	42.7	NA
Marks [1998] [65]	1994–1996	Canada and United States	1	3120	1185	38	47	NA	312	10	NA
Marks [1995] [66]	1992–1994	Canada and United States	1	3549	1349	38	46	NA	351	9.9	NA
Navarro‐Triviño [2024] [139]	2018–2020	Spain	2	8760	NA	NA	NA	NA	NA	NA	NA
Nielsen [2001] [113]	1990–1998	Denmark	NA	759	329	43.3	NA	NA	NA	NA	NA
Noë [2022] [58]	2010–2019	Belgium	1/2	329	136	41	10.9	10.9	179	54.4	78
Odhav [2012] [153]	1994–2010	United States	1	1905	692	36.3	NA	NA	NA	NA	NA
Pesonen [2015] [37]	2002–2010	11 European countries	NA	44 277	16 968	38.3	41	40.5	NA	NA	NA
Piapan [2024] [61]	1996–2021	Italy	1	30 629	9935	32.4	NA	43.8 ± 17.2	2768	10.1	NA
Piaserico [2004] [18]	1997–2001	Italy	NA	1444	464	32.1	72.7	72.7 ± 5.7	NA	NA	NA
Pontén [2013] [166]	2009–2010	10 countries	1&2	3591	1196	33.3	NA	NA	NA	NA	NA
Pontén [2016] [133]	2012–2014	Sweden	2	2165	736	34	NA	NA	NA	NA	NA
Prodi [2016] [39]	1996–2012	Italy	NA	23 774	7718	32.5	NA	43.1 ± 17.0	NA	NA	NA
Puangpet [2023] [106]	NA	Thailand	1	124	63	50.8	8	NA	50	40.3	19
Racheva [2006] [119]	NA	Bulgaria	NA	210	NA	NA	NA	NA	NA	NA	NA
Rastogi [2018] [42]	2014–2017	United States	1	502	121	24.1	NA	47.1 ± 14.8	108	21.5	72
Rietschel [2002] [68]	1998–2000	Canada and United States	1	5839	NA	NA	NA	NA	428	7.3	NA
Rodrigues [2012] [154]	2003–2010	Brazil	1	1406	426	30.3	NA	NA	NA	NA	NA
Schnuch [1998] [67]	1990–1994	3 European countries	1	41 621	15 469	37.2	NA	NA	8572	20.6	NA
Schnuch [2008] [121]	1996–2007	3 European countries	1	NA	NA	NA	NA	NA	NA	NA	NA
Schnuch [2011] [126]	1996–2009	3 European countries	1	121 558	45 832	37.7	NA	NA	NA	NA	NA
Sharma [1998] [161]	NA	India	1	200	122	61	39.8	NA	NA	NA	NA
Shenoi [1994] [160]	1992–1993	India	1	212	147	69.3	NA	NA	NA	NA	NA
Silva [2020] [51]	2015–2017	Brazil	2	267	72	27.1	NA	43.0 ± 16.0	NA	NA	NA
Silverberg [2021] [102]	2001–2016	Canada and United States	1&2	38 482	12 609	32.8	NA	NA	9136	23.7	NA
Silverberg [2022] [149]	2001–2018	Canada and United States	1&2	41 699	NA	NA	NA	NA	NA	NA	NA
Simonsen [2014] [87]	2003–2011	Denmark	NA	2594	884	34.1	NA	NA	1162	44.8	NA
Simonsen [2018] [43]	2012–2016	Denmark	1	1573	568	36.1	12.5	12.5 ± 4.0	823	52.3	188
Simonsen [2018] [94]	2014–2017	Denmark	1	100	39	39	9.8	NA	100	100	48
Slodownik [2024] [60]	2019–2022	Israel	2	2086	685	33	NA	44.4 ± 0.8	396	19	NA
Storrs [1989] [16]	1984–1985	Canada and United States	2	2519	1103	43.8	41	42.9	218	18.2	NA
Sukakul [2019] [49]	2006–2018	Thailand	2	2803	668	23.8	NA	42.4 ± 15.5	NA	NA	NA
Søgaard [2025] [63]	2014–2023	Denmark	1/2	6435	2018	31.4	NA	47.8 ± 16.9	1469	22.8	116
Tagka [2019] [48]	2014–2016	Greece	1^b^	1978	619	31.3	NA	45.9 ± 18.6	692	35	NA
Tam [2020] [50]	2007–2016	United States	1	2373	632	26.6	47.7	47.7 ± 16.9	695	29.3	NA
Teo [2019] [96]	1984–2014	United Kingdom	NA	46 250	19 189	41.5	NA	NA	14 732	31.9	13 592
Thyssen [2012] [83]	1984–2010	Denmark	1	15 641	5681	36.3	NA	NA	1218	7.8	334
Thyssen [2010] [77]	1985–2008	Denmark	1	18 179	6590	35.7	NA	NA	2436	13.4	NA
Toholka [2015] [90]	2001–2010	Australia	1	5281	1865	35	41	NA	1576	30	NA
Tunca [2019] [46]	2001–2015	Turkey	NA	673	296	44	29.4	29.4 ± 10.3	NA	NA	NA
Uter [2021] [101]	2007–2012	Germany	1&0.18^c^	2213	797	36	NA	NA	369	16.7	NA
Uter [2022] [5]	2019–2020	13 European countries	1&2&0.18^c^	22 474	NA	NA	NA	NA	NA	NA	NA
Uter [2002] [114]	1994–2000	3 European countries	1	67 915	NA	NA	NA	NA	NA	NA	NA
Uter [2012] [128]	2007–2008	11 European countries	1	25 181	NA	NA	NA	NA	NA	NA	NA
Uter [2005] [72]	2002–2003	9 European countries	1	10 511	3899	37.1	NA	NA	1892	18	NA
Veverka [2018] [41]	2011–2015	United States	1	2582	843	32.7	NA	53.4 ± 17.4	NA	NA	NA
Wang [2005] [19]	2001–2003	China	1	488	120	24.6	NA	40.5 ± 16.4	9	1.8	NA
Warshaw [2018] [93]	2000–2014	Canada and United States	1&2	32 945	11 070	33.6	NA	NA	7433	22.6	NA
Warshaw [2012] [85]	1994–2008	Canada and United States	1	31 942	11 457	35.9	NA	NA	18 362	57.5	NA
Warshaw [2015] [38]	2011–2012	Canada and United States	1	4238	1329	31.4	50	48	1188	28	NA
Warshaw [2013] [34]	2009–2010	Canada and United States	1	4308	1381	32.1	48.4	48.4	982	22.8	NA
Wee [2022] [57]	2007–2017	Singapore	1	4903	2044	41.7	NA	40.1 ± 16.3	2529	51.6	1280
Wentworth [2014] [36]	2006–2010	United States	1	3115	1070	34.3	NA	54.9 ± 18.3	NA	NA	NA
Wetter [2005] [21]	1998–2000	United States	1	1324	455	34.4	54.7	NA	NA	NA	NA
Whitehouse [2021] [136]	2015–2018	11 European countries	1&2	NA	NA	NA	NA	NA	NA	NA	NA
Yin [2011] [31]	2004–2009	China	1	2758	1136	41.2	38.5	38.5 ± 12.4	215	7.8	NA
Yu [2017] [40]	2009–2014	South Korea	1^b^	330	100	30.3	40.6	40.6 ± 16.2	NA	NA	NA
Yücel [2021] [55]	2017–2018	Turkey	NA	80	35	43.7	7.37	7.4 ± 3.8	NA	NA	NA
Zug [2008] [75]	2001–2004	Canada and United States	1	10 061	NA	NA	NA	NA	1820	18.1	1084
Zug [2009] [76]	2005–2006	Canada and United States	1	4454	1667	37.4	NA	NA	960	21.6	NA
de Groot [2010] [124]	1994–2009	Netherlands	0.18^c^	6503	2523	39	NA	NA	NA	NA	NA
de Groot [1985] [109]	NA	Netherlands	1	179	34	19	NA	NA	NA	NA	NA
Çalka [2011] [142]	NA	Turkey	1	115	61	53	33.42	NA	NA	NA	NA
Özel [2013] [35]	2011–2012	Turkey	NA	168	74	44	34.1	34.1 ± 14.9	NA	NA	NA

Abbreviations: n, number; NA, not applicable.

^a^ If nothing else denoted, then the vehicle is aqueous.

^b^ Petrolatum.

^c^ mg/cm^2^.

### 3.2 Quantitative Assessment

#### 3.2.1 Contact Allergy to Formaldehyde and Releasers

The pooled prevalence of contact allergy to formaldehyde in 1 085 443 patients was 2.88% (95% CI 2.55–3.24, *n* = 148 studies [4, 5, 6, 8, 16, 17, 18, 19, 20, 21, 22, 23, 24, 26, 27, 28, 29, 30, 31, 32, 34, 35, 36, 37, 38, 39, 40, 41, 42, 43, 44, 45, 46, 47, 48, 49, 50, 51, 53, 54, 55, 56, 57, 58, 59, 60, 61, 62, 63, 64, 65, 66, 67, 68, 69, 70, 71, 72, 73, 74, 75, 76, 77, 78, 79, 81, 82, 83, 84, 85, 86, 87, 88, 89, 90, 91, 92, 93, 94, 96, 97, 99, 100, 101, 102, 103, 104, 105, 106, 107, 108, 109, 111, 112, 113, 114, 116, 117, 118, 119, 120, 121, 122, 123, 124, 125, 126, 127, 128, 129, 130, 131, 132, 133, 134, 135, 136, 137, 138, 139, 140, 141, 142, 143, 144, 145, 146, 147, 148, 149, 150, 151, 153, 154, 155, 156, 157, 158, 159, 160, 161, 162, 163, 164, 165, 166, 167, 168]). There was a high level of heterogeneity with an I^2^ of 98.9% (95% CI 98.8–98.9). The clinical relevance of PPTs was 41.57% (95% CI 32.76–50.95, *n* = 43 studies [6, 8, 16, 20, 27, 28, 31, 34, 36, 38, 41, 43, 44, 56, 60, 65, 66, 71, 72, 75, 76, 84, 85, 86, 87, 90, 93, 94, 97, 100, 106, 114, 122, 131, 136, 138, 139, 140, 143, 149, 159, 162, 164]). Among the formaldehyde releasers, 2‐bromo‐2‐nitropropane‐1,3‐diol showed the highest pooled prevalence at 2.76% (95% CI 2.19–3.47, *n* = 68 studies [4, 5, 6, 7, 8, 16, 19, 21, 22, 25, 29, 31, 33, 34, 36, 38, 41, 42, 43, 44, 45, 50, 52, 55, 58, 59, 64, 65, 66, 67, 68, 71, 74, 75, 76, 77, 80, 82, 85, 86, 87, 89, 90, 91, 93, 96, 98, 102, 109, 111, 115, 117, 120, 121, 126, 128, 130, 135, 136, 138, 139, 144, 145, 149, 151, 162, 163, 164]). Quaternium‐15 showed a prevalence of 1.89% (95% CI 1.57–2.28, *n* = 117 studies [4, 5, 6, 7, 8, 16, 17, 18, 21, 22, 23, 24, 25, 26, 28, 30, 33, 34, 35, 36, 37, 38, 40, 41, 42, 43, 44, 45, 48, 49, 50, 51, 52, 53, 54, 56, 58, 59, 61, 62, 64, 65, 66, 67, 68, 69, 71, 72, 73, 75, 76, 77, 78, 81, 82, 83, 84, 85, 86, 87, 88, 89, 90, 92, 93, 94, 96, 100, 102, 103, 105, 107, 109, 111, 113, 116, 117, 118, 120, 121, 122, 123, 124, 125, 126, 128, 130, 131, 132, 135, 136, 137, 138, 139, 140, 141, 142, 143, 144, 145, 146, 148, 149, 150, 151, 152, 153, 154, 155, 158, 160, 161, 162, 163, 164, 165, 168]) and the highest clinical relevance at 55.67% (95% CI 42.79–67.84, *n* = 40 studies [6, 7, 8, 16, 25, 28, 34, 36, 38, 41, 43, 44, 56, 65, 66, 71, 72, 75, 76, 84, 85, 86, 87, 90, 93, 94, 100, 125, 131, 132, 136, 138, 139, 140, 141, 143, 149, 160, 162, 164]). The prevalence rate for diazolidinyl urea was 1.42% (95% CI 1.13–1.79, *n* = 71 studies [4, 5, 6, 7, 8, 16, 21, 22, 25, 30, 33, 34, 36, 38, 41, 42, 43, 44, 45, 49, 50, 52, 55, 59, 65, 66, 67, 68, 71, 75, 76, 77, 82, 84, 85, 86, 87, 88, 89, 90, 93, 94, 95, 96, 97, 98, 103, 107, 110, 111, 117, 120, 121, 122, 126, 128, 130, 131, 132, 135, 136, 137, 138, 139, 144, 145, 151, 162, 163, 164, 168]), DMDM hydantoin 1.37% (95% CI 1.07–1.76, *n* = 42 studies [4, 8, 16, 21, 22, 25, 30, 33, 34, 36, 38, 41, 42, 45, 50, 52, 59, 65, 66, 67, 68, 75, 76, 77, 82, 85, 86, 89, 90, 93, 107, 114, 115, 121, 126, 130, 135, 136, 151, 162, 163, 164]), and imidazolidinyl urea 1.20% (95% CI 0.97–1.47, *n* = 76 studies [4, 5, 6, 7, 8, 16, 19, 21, 22, 25, 28, 29, 30, 31, 33, 34, 36, 38, 40, 41, 42, 43, 44, 45, 49, 50, 52, 55, 59, 64, 65, 66, 67, 68, 71, 74, 75, 76, 77, 82, 84, 85, 86, 87, 89, 90, 93, 94, 96, 98, 103, 107, 111, 115, 117, 120, 121, 122, 126, 128, 130, 131, 132, 135, 136, 138, 139, 141, 144, 145, 150, 151, 162, 163, 164, 168]) (Table 2).

**TABLE 2:** Proportion of positive patch tests to formaldehyde and formaldehyde releasers in all patients.

Allergen	Studies, *n*	Patients undergoing patch testing, *n*	Proportion of PPTs, % [95% CI]	Tau^2^ [95% CI]	I^2^ [95% CI] (%)	Clinical relevance studies, *n*	Clinical relevance (%) [95% CI]
**Formaldehyde**	148	1 085 443	2.88 [2.55; 3.24]	0.50 [0.53; 0.99]	98.9 [98.8; 98.9]	43	41.57 [32.76; 50.95]
**Quaternium‐15**	117	894 513	1.89 [1.57; 2.28]	0.97 [0.82; 1.78]	99.5 [99.4; 99.5]	40	55.67 [42.79; 67.84]
**2‐Bromo‐2‐nitropropane‐1,3‐diol**	68	267 085	2.76 [2.19; 3.47]	0.44 [0.53; 2.04]	99.3 [99.3; 99.4]	31	31.50 [21.18; 44.04]
**Imidazolidinyl urea**	76	554 265	1.20 [0.97; 1.47]	0.76 [0.54; 1.34]	98.6 [98.4; 98.7]	34	33.32 [25.25; 43.12]
**Diazolidinyl urea**	71	544 564	1.42 [1.13; 1.79]	0.92 [0.61; 1.62]	99.3 [99.2; 99.3]	31	31.91 [24.38; 40.50]
**DMDM hydantoin**	42	412 788	1.37 [1.07; 1.76]	0.67 [0.39; 1.24]	98.7 [98.5; 98.8]	20	26.14 [18.78; 35.14]

Abbreviations: CI, confidence interval; DMDM hydantoin, 1,3‐dimethylol‐5,5‐dimethylhydantoin; n, number; PPTs, positive patch tests.

#### 3.2.2 Contact Allergy According to Concentration

No significant difference was found for formaldehyde (*p* = 0.77) between 1% aq with a prevalence of 3.04% (95% CI 2.51–3.68, *n* = 86 studies [4, 5, 6, 8, 17, 19, 21, 22, 23, 24, 28, 29, 30, 31, 32, 34, 36, 38, 41, 42, 43, 45, 50, 57, 61, 65, 66, 67, 68, 69, 70, 71, 72, 73, 75, 76, 77, 78, 82, 83, 84, 85, 86, 88, 90, 91, 93, 94, 101, 102, 106, 109, 112, 114, 116, 117, 120, 121, 122, 125, 126, 127, 128, 129, 130, 131, 134, 136, 141, 142, 146, 149, 150, 151, 153, 154, 157, 160, 161, 162, 163, 164, 165, 166, 167, 168]) and 2% aq with a prevalence of 3.22% (95% CI 2.30–4.48, *n* = 29 studies [4, 5, 6, 8, 16, 47, 49, 51, 54, 56, 60, 93, 100, 102, 103, 107, 108, 122, 129, 133, 136, 138, 139, 140, 147, 149, 156, 166, 168]). Quaternium‐15 had a higher prevalence of 5.83% for 2% pet (95% CI 4.37–7.73, *n* = 24 studies [8, 16, 17, 21, 34, 38, 42, 49, 50, 51, 59, 65, 66, 75, 76, 85, 86, 93, 102, 103, 149, 151, 153, 168]) than for 1% pet, which had a prevalence of 1.31% (95% CI 1.06–1.61, *n* = 68 studies [4, 5, 6, 7, 21, 22, 23, 24, 25, 28, 30, 33, 36, 40, 41, 43, 44, 45, 48, 52, 54, 56, 58, 61, 67, 68, 69, 71, 72, 73, 77, 78, 82, 83, 84, 88, 90, 92, 94, 100, 105, 107, 116, 117, 120, 121, 122, 126, 128, 130, 131, 135, 136, 137, 138, 139, 140, 141, 142, 146, 150, 152, 158, 160, 162, 163, 164, 165]). There was a significant difference (*p* < 0.01). Significant differences were also observed across the tested subgroups for imidazolidinyl urea (*p* = 0.01), diazolidinyl urea (*p* < 0.01), and DMDM hydantoin (*p* < 0.01). For diazolidinyl urea, the significant difference appears to be primarily associated with the vehicle rather than the concentration. A higher proportion of positive reactions was observed in aqueous solutions (2.40% and 2.73%) compared to petrolatum (0.94% and 0.93%). No significant difference was found for 2‐bromo‐2‐nitropropane‐1,3‐diol (*p* = 0.09) (Table 3).

**TABLE 3:** Proportion of positive patch tests to formaldehyde and formaldehyde releasers according to concentration.

Allergen	Concentration	Studies, *n*	Patients undergoing patch testing, *n*	Proportion of PPTs, % [95% CI]	Tau^2^	I^2^ (%)	Test for subgroup differences (random effects): Chi‐squared *p*‐value
**Formaldehyde**	1 aq	86	842 126	3.04 [2.51; 3.68]	0.60	99.5	0.77
	2 aq	29	135 772	3.22 [2.30; 4.48]	0.50	98.5	
**Quaternium‐15**	1 pet	68	581 145	1.31 [1.06; 1.61]	1.40	99.3	< 0.01
	2 pet	24	207 486	5.83 [04.37; 7.73]	0.08	97.5	
**2‐Bromo‐2‐nitropropane‐1,3‐diol**	0.25 pet	21	41 556	0.97 [0.63; 1.51]	0.41	88.7	0.09
	0.5 pet	37	391 453	1.48 [01.16; 1.89]	0.28	97.8	
**Imidazolidinyl urea**	2 aq	19	114 507	1.52 [1.26; 1.84]	0.04	78.1	0.01
	2 pet	57	442 718	1.04 [0.82; 1.32]	0.54	97.7	
**Diazolidinyl urea**	1 aq	14	102 404	2.40 [2.00; 2.88]	0.03	82.6	< 0.01
	1 pet	19	30 167	0.94 [0.55; 1.59]	0.09	65.9	
	2 aq	6	134 818	2.73 [2.37; 3.15]	0.03	81.1	
	2 pet	36	296 239	0.93 [0.71; 1.23]	1.98	99.5	
**DMDM hydantoin**	1 aq	21	234 954	1.65 [1.44; 1.90]	0.05	86.3	< 0.01
	2 aq	23	232 314	0.85 [0.62; 1.18]	0.46	95.8	

Abbreviations: aq, aqua; CI, confidence interval; DMDM hydantoin, 1,3‐dimethylol‐5,5‐dimethylhydantoin; n, number; pet, petrolatum; PPTs, positive patch tests.

#### 3.2.3 Contact Allergy in Patients With and Without AD

A comparison was made between 31 361 patients with AD and 85 553 patients without AD for formaldehyde allergy (*n* = 10 studies [43, 57, 70, 79, 81, 83, 89, 96, 102, 107]). The pooled prevalence rates were 2.65% (95% CI 1.19–5.77) for patients with AD and 2.85% (95% CI 1.25–6.34) for patients without AD, with no significant difference (*p* = 0.89). There were no significant differences for quaternium‐15 (*p* = 0.72), 2‐bromo‐2‐nitropropane‐1,3‐diol (*p* = 0.54), imidazolidinyl urea (*p* = 0.82), or diazolidinyl urea (*p* = 0.81). However, a significant difference was noted for DMDM hydantoin, with a prevalence of 1.73% (95% CI 0.97–3.08) in 318 patients with ad and 0.57% (95% CI 0.00–81.86) in 1950 patients without AD (*p* = 0.03). This result should be interpreted with caution due to the small number of studies reporting on this topic (Table 4).

**TABLE 4:** Comparison analysis of dermatitis patients with versus without AD.

Allergen	Studies [n]	Non‐AD patients undergoing patch testing [n]	Non‐AD proportion of PPTs [95% CI] (%)	AD patients undergoing patch testing [n]	AD proportion of PPTs [95% CI] (%)	Test for subgroup differences (random effects): Chi‐squared *p*‐value
**Formaldehyde**	10	85 553	2.85 [1.25; 6.34]	31 361	2.65 [1.19; 5.77]	0.89
**Quaternium‐15**	7	77 338	2.19 [0.76; 6.19]	24 970	1.76 [0.58; 5.21]	0.72
**2‐Bromo‐2‐nitropropane‐1,3‐diol**	4	28 304	0.86 [0.23; 3.22]	13 340	1.21 [0.39; 3.65]	0.54
**Imidazolidinyl urea**	4	28 841	0.65 [0.27; 1.56]	12 966	0.65 [0.27; 1.56]	0.82
**Diazolidinyl urea**	5	22 946	1.25 [0.36; 4.22]	10 932	1.49 [0.28; 7.52]	0.81
**DMDM hydantoin**	2	1950	0.57 [0.00; 81.86]	318	1.73 [0.97; 3.08]	0.03

Abbreviations: AD, atopic dermatitis; CI, confidence interval; DMDM hydantoin, 1,3‐dimethylol‐5,5‐dimethylhydantoin; n, number; PPTs, positive patch tests.

#### 3.2.4 Contact Allergy According to Geographical Region

The highest rates of formaldehyde contact allergy were found in North America with a prevalence of 6.8% (95% CI 5.7–8.0, *n* = 31 studies [8, 16, 17, 21, 24, 27, 34, 36, 38, 41, 42, 50, 53, 64, 65, 66, 68, 75, 76, 79, 81, 85, 86, 93, 102, 147, 148, 149, 150, 151, 153]). North America also reported the highest rates of quaternium‐15, at 7.5% (95% CI 6.7–8.3, *n* = 30 studies [8, 16, 17, 21, 24, 25, 34, 36, 38, 41, 42, 50, 53, 64, 65, 66, 68, 75, 76, 81, 85, 86, 93, 102, 148, 149, 150, 151, 152, 153]). The rates for the other releasers were 2‐bromo‐2‐nitropropane‐1,3‐diol at 2.4% (95% CI 2.1–2.8, *n* = 22 studies [8, 16, 21, 25, 34, 36, 38, 41, 42, 50, 64, 65, 66, 68, 75, 76, 85, 86, 93, 102, 149, 151]), imidazolidinyl urea at 2.0% (95% CI 1.8–2.3, *n* = 21 studies [8, 16, 21, 25, 34, 36, 38, 41, 42, 50, 64, 65, 66, 68, 75, 76, 85, 86, 93, 150, 151]), diazolidinyl urea at 2.6% (95% CI 2.3–2.9, *n* = 19 studies [8, 16, 21, 25, 34, 36, 38, 41, 42, 50, 65, 66, 68, 75, 76, 85, 86, 93, 151]), and DMDM hydantoin at 1.6% (95% CI 1.4–1.9, *n* = 19 studies [8, 16, 21, 25, 34, 36, 38, 41, 42, 50, 65, 66, 68, 75, 76, 85, 86, 93, 151]). The lowest rate of formaldehyde contact allergy was found in Southeast Asia at 1.8% (95% CI 0.7–4.3, *n* = 11 studies [30, 44, 49, 57, 59, 103, 106, 160, 161, 162, 163]). In Europe, the prevalence was 2.3% (95% CI 2.0–2.6, *n* = 65 studies [4, 5, 6, 18, 28, 32, 37, 39, 43, 48, 54, 58, 61, 63, 67, 70, 71, 72, 73, 77, 78, 82, 83, 84, 87, 88, 89, 91, 92, 94, 96, 101, 105, 107, 108, 109, 111, 112, 113, 114, 116, 117, 118, 119, 120, 121, 122, 123, 124, 125, 126, 127, 128, 129, 130, 131, 132, 133, 134, 135, 136, 137, 138, 139, 140]) (Table 5).

**TABLE 5:** Proportion of positive patch tests to formaldehyde and formaldehyde releasers according to geographical region.

Europe		Middle East		North America		South America		East Asia		Southeast Asia		Oceania		Africa	
Studies [n]	Proportion of PPTs (95% CI) [%]	Studies [n]	Proportion of PPTs (95% CI) [%]	Studies [n]	Proportion of PPTs (95% CI) [%]	Studies [n]	Proportion of PPTs (95% CI) [%]	Studies [n]	Proportion of PPTs (95% CI) [%]	Studies [n]	Proportion of PPTs (95% CI) [%]	Studies [n]	Proportion of PPTs (95% CI) [%]	Studies [n]	Proportion of PPTs (95% CI) [%]
**Formaldehyde**															
65	2.3 [2.0; 2.6]	16	1.9 [1.3; 2.7]	31	6.8 [5.7; 8.0]	3	5.4 [1.4; 18.4]	11	4.2 [2.4; 7.4]	11	1.8 [0.7; 4.3]	4	4.1 [2.2; 7.5]	4	5.3 [0.8; 27.3]
**Quaternium‐15**															
52	1.0 [0.9; 1.1]	12	1.0 [0.6; 1.6]	30	7.5 [6.7; 8.3]	3	1.8 [0.7; 4.5]	5	1.8 [0.5; 6.7]	9	1.4 [1.2; 1.6]	2	3.1 [1.1; 8.9]	3	1.5 [0.2; 8.2]
**2‐Bromo‐2‐nitropropane‐1,3‐diol**															
29	0.8 [0.6; 1.0]	4	1.7 [0.1; 21.9]	22	2.4 [2.1; 2.8]	NA	NA	7	1.5 [0.6; 4.0]	4	0.8 [0.2; 2.8]	2	1.0 [0.3; 4.0]	NA	NA
**Imidazolidinyl urea**															
32	0.6 [0.5; 0.7]	5	1.8 [0.4; 7.2]	21	2.0 [1.8; 2.3]	NA	NA	8	2.0 [1.0; 3.9]	7	1.0 [0.8; 1.4]	2	1.9 [1.5; 2.6]	NA	NA
**Diazolidinyl urea**															
33	0.7 [0.6; 0.9]	4	0.9 [0.1; 6.8]	19	2.6 [2.3; 2.9]	NA	NA	4	2.4 [0.7; 7.9]	7	1.2 [0.6; 2.3]	3	2.4 [1.3; 4.4]	NA	NA
**DMDM hydantoin**															
13	0.5 [0.4; 0.6]	1	0.6 [0.2; 2.6]	19	1.6 [1.4; 1.9]	NA	NA	3	1.6 [1.2; 2.1]	4	1.0 [0.1; 6.7]	2	2.4 [0.6; 9.4]	NA	NA

Abbreviations: CI, confidence interval; DMDM hydantoin, 1,3‐dimethylol‐5,5‐dimethylhydantoin; n, number; NA, not applicable; PPTs, positive patch tests.

#### 3.2.5 Contact Allergy According to Study Period

The pooled prevalence of formaldehyde was 3.04% (95% CI 2.63–3.53, *n* = 127 studies [4, 6, 8, 16, 17, 18, 19, 20, 21, 22, 23, 24, 26, 27, 28, 29, 30, 31, 32, 34, 35, 36, 37, 38, 39, 40, 41, 42, 43, 44, 45, 46, 47, 48, 49, 50, 51, 53, 54, 55, 56, 57, 58, 62, 64, 65, 66, 67, 68, 69, 70, 71, 72, 73, 74, 75, 76, 77, 78, 81, 82, 83, 84, 85, 86, 87, 88, 89, 90, 91, 92, 93, 94, 96, 97, 99, 100, 101, 102, 103, 104, 108, 111, 112, 113, 114, 116, 117, 118, 120, 121, 122, 123, 124, 126, 127, 128, 129, 130, 131, 132, 133, 134, 136, 137, 141, 143, 144, 147, 148, 149, 150, 151, 153, 154, 155, 156, 157, 158, 159, 160, 162, 164, 165, 166, 167, 168]) before 2019 and 1.77% (95% CI 0.63–4.87, *n* = 3 studies [5, 60, 138]) after 2019. The pooled prevalence for quaternium‐15 was 2.02% (95% CI 1.63–2.50, *n* = 101 studies [4, 6, 7, 8, 16, 17, 18, 21, 22, 23, 24, 25, 26, 28, 30, 33, 34, 35, 36, 37, 38, 40, 41, 42, 43, 44, 45, 48, 49, 50, 51, 52, 53, 54, 56, 58, 62, 64, 65, 66, 67, 68, 69, 71, 72, 73, 75, 76, 77, 78, 81, 82, 83, 84, 85, 86, 87, 88, 89, 90, 92, 93, 94, 96, 100, 102, 103, 111, 113, 116, 117, 118, 120, 121, 122, 123, 124, 126, 128, 130, 131, 132, 136, 137, 141, 143, 144, 148, 149, 150, 151, 152, 153, 154, 155, 158, 160, 162, 164, 165, 168]) before 2019 and 0.80% (95% CI 0.24–2.62, *n* = 2 studies [5, 138]) after 2019. The differences between these time periods were not significant for formaldehyde (*p* = 0.15) or quaternium‐15 (*p* = 0.18). However, these results should be interpreted with caution due to the small number of studies reporting on this after 2019 (Table 6).

**TABLE 6:** Proportion of positive patch tests to formaldehyde and quaternium‐15 according to study period before and after 2019.

Allergen	Study period [year]	Studies [*n*]	Proportion of PPTs (95% CI) [%]	Test for subgroup differences (random effects): Chi‐squared *p*‐value
**Formaldehyde**	< 2019	127	3.04 [2.63; 3.53]	0.15
	> 2019	3	1.77 [0.63; 4.87]	
**Quaternium‐15**	< 2019	101	2.02 [1.63; 2.50]	0.18
	> 2019	2	0.80 [0.24; 2.62]	

Abbreviations: CI, confidence interval; *n*, number; PPTs, positive patch tests.

#### 3.2.6 Contact Allergy According to Age

The prevalence of contact allergy to formaldehyde was compared between children and adults. The pooled prevalence was 2.96% (95% CI 1.30–6.58, *n* = 12 studies [27, 32, 43, 53, 55, 58, 87, 94, 97, 106, 107, 144]) for children and 2.61% (95% CI 1.41–4.77, *n* = 10 studies [18, 26, 42, 63, 78, 125, 138, 140, 143, 145]) for adults, with no significant difference (*p* = 0.79). Similarly, no significant differences were found for quaternium‐15 (*p* = 0.83), 2‐bromo‐2‐nitropropane‐1,3‐diol (*p* = 0.42), imidazolidinyl urea (*p* = 0.35), diazolidinyl urea (*p* = 0.14), or DMDM hydantoin (*p* = 0.86) (Table 7).

**TABLE 7:** Proportion of positive patch tests in children and adults.

Allergen	Age	Studies [*n*]	Proportion of PPTs (95% CI) [%]	Test for subgroup differences (random effects): Chi^2^ *p*‐value
**Formaldehyde**	Children	12	2.96 [1.30; 6.58]	0.79
	Adults	10	2.61 [1.41; 4.77]	
**Quaternium‐15**	Children	8	0.92 [0.32; 2.48]	0.83
	Adults	9	1.01 [0.63; 1.62]	
**2‐Bromo‐2‐nitropropane‐1,3‐diol**	Children	6	1.65 [0.2; 12.4]	0.42
	Adults	3	0.93 [0.24; 3.46]	
**Imidazolidinyl urea**	Children	7	0.96 [0.28; 3.2]	0.35
	Adults	3	0.58 [0.21; 1.56]	
**Diazolidinyl urea**	Children	8	0.95 [0.44; 2.0]	0.14
	Adults	3	0.52 [0.18; 1.47]	
**DMDM hydantoin**	Children	2	0.66 [0.23; 1.91]	0.86
	Adults	1	0.60 [0.19; 1.84]	

Abbreviations: CI, confidence interval; DMDM hydantoin, 1,3‐dimethylol‐5,5‐dimethylhydantoin; *n*, number; PPTs, positive patch tests.

## 4 Discussion

This systematic review and meta‐analysis of 158 studies encompasses 1 347 638 dermatitis patients. The overall rate of contact allergy to formaldehyde in dermatitis patients was 2.88% (95% CI 2.55–3.24). Prevalence rates of its common releasers were as follows: 2‐bromo‐2‐nitropropane‐1,3‐diol (2.76%), quaternium‐15 (1.89%), diazolidinyl urea (1.42%), DMDM hydantoin (1.37%), and imidazolidinyl urea (1.20%).

Compared with other preservatives such as methylchloroisothiazolinone/methylisothiazolinone (MCI/MI; 6.91%), methylisothiazolinone (MI; 7.57%), and methyldibromo glutaronitrile (5.65%) [5], the prevalence of contact allergy to formaldehyde and its releasers is lower. The clinical relevance of a positive patch test to formaldehyde was 41.57%. Quaternium‐15 showed the highest clinical relevance at 55.67%. Formaldehyde and its releasers have a lower clinical relevance compared to MCI/MI (69.12%) and MI (77.52%), but a higher clinical relevance than methyldibromo glutaronitrile (10.80%) [139]. However, studies reporting on clinical relevance are relatively limited in number compared to studies reporting frequencies of PPTs. Consistent reporting is important in future studies, as with other allergens [169, 170].

We found no significant difference (*p* = 0.89) in the prevalence of formaldehyde contact allergy between patients with AD (2.65%) and those without AD (2.85%). The same was true for quaternium‐15, 2‐bromo‐2‐nitropropane‐1,3‐diol, imidazolidinyl urea, and diazolidinyl urea. However, a significant difference was noted for DMDM hydantoin (*p* = 0.03). This sub‐analysis was limited to two studies, which restricts the generalizability of the findings. Nevertheless, the overall finding of no difference in sensitivity to formaldehyde and releasers is consistent with other studies [171, 172] and suggests that AD status is not a risk factor for formaldehyde sensitization.

Our sub‐analysis of age showed no significant differences (*p* = 0.79) in formaldehyde contact allergy between children (2.96%) and adults (2.61%), or among any of the releasers. These results suggest that sensitization occurs early in life from sources common to both populations, such as personal care products, cosmetics, and household items. Furthermore, the prevalence of contact allergies to formaldehyde, imidazolidinyl urea, and diazolidinyl urea among children in the current study is higher than that reported in a recent meta‐analysis of children [169]. However, the latter only included data from studies conducted between 2010 and 2024.

Significant differences in formaldehyde sensitization rates were found between geographic regions. The highest rate was found in North America (6.8%). This rate is nearly three times higher than the rates found in Europe (2.3%) and Southeast Asia (1.8%). This disparity likely reflects differences in regulatory policy rather than exposure patterns alone. While the EU prohibited the use of formaldehyde and quaternium‐15 as cosmetic ingredients in 2019, these ingredients are still permitted in North America. The lower prevalence in Europe suggests that the region's long‐standing regulatory approach has been an effective public health intervention.

Regarding temporal trends, our findings show that the pooled prevalence of formaldehyde decreased from 3.04% in studies conducted before 2019 to 1.77% in studies conducted after 2019. A similar decrease was observed for quaternium‐15, dropping from 2.02% to 0.80% during the same period. However, these differences were not statistically significant for formaldehyde (*p* = 0.15) or quaternium‐15 (*p* = 0.18). The post‐2019 analysis is also based on a small number of studies. The lack of a statistically significant decline may indicate that the full effects of the 2019 legislation have yet to be reflected in the literature.

A key epidemiological factor not considered is the high rate of co‐sensitization between formaldehyde and its releasers. Interpreting a positive reaction to a formaldehyde releaser clinically is complex because it may be caused by primary sensitization to formaldehyde, specific sensitization to the releaser molecule itself, or both. The amount of formaldehyde released by releasers varies, and this is often reflected in the degree of co‐sensitization [173]. For instance, a positive reaction to quaternium‐15 is highly associated with contact allergy to formaldehyde [111, 124]. Imidazolidinyl urea, diazolidinyl urea, and DMDM hydantoin are also associated with formaldehyde [174]. Conversely, 2‐bromo‐2‐nitropropane‐1,3‐diol is less frequently associated with formaldehyde allergy, which suggests that it may act as an independent sensitizer [135, 175]. Clinicians must consider the underlying complexity of concomitant reactions when interpreting patch test results.

From a diagnostic standpoint, our findings provide a complex picture of the testing concentrations. There was no significant difference in detection rates between the 1% and 2% formaldehyde aqueous (aq) solutions (3.04% and 3.22% respectively, *p* = 0.77). However, this overall result contrasts with several studies that directly compared the two concentrations and concluded that 2% aq detects significantly more positive reactions [122, 166, 168]. Our pooled finding is more consistent with other studies that also found no significant difference [176]. This may be due to methodological differences of patch testing compared to the experimental design. Adding to the complexity, other studies suggest that even 2% aq may be inadequate for identifying sensitization to formaldehyde releasers [136]. For quaternium‐15, the 2% pet detected a significantly (*p* < 0.01) higher prevalence (5.83%) than the 1% pet (1.31%). Similar significant differences were found for other releasers. However, as with diazolidinyl urea, the vehicle may influence diagnostic outcomes more than the concentration itself. In our analysis, aqueous solutions appeared to be associated with a higher proportion of positive reactions than petrolatum preparations. These findings suggest that using petrolatum vehicles or 1% petrolatum to test releasers could result in an underdiagnosis of sensitization to these allergens.

### 4.1 Strengths and Limitations

The primary strength of this meta‐analysis is its large scale, encompassing 158 studies with over 1.3 million patients with dermatitis. This provides a generalizable estimate of the prevalence of contact allergy to formaldehyde and its releasers. Furthermore, the extensive sub‐analyses offer valuable epidemiological insights. However, the findings are subject to limitations.

There was a high level of statistical heterogeneity (I^2^ = 98.9%), indicating substantial variability across the included studies. This may reflect differences in clinical populations, geographic regions, and patch test methodologies, and suggests that the pooled estimates should be interpreted with caution. However, the articles generally scored a low risk of bias based on the AXIS assessment in Supplementary Table 2. Additionally, the scarcity of studies published since the 2019 EU regulation limits the validity of our temporal trend analysis. Lastly, we excluded general population data to align with our primary aim of focusing on patients with dermatitis. Comparing these data was deemed outside the scope of this study and would introduce significant methodological heterogeneity.

### 4.2 Gaps in Knowledge

Significant knowledge gaps exist primarily due to a lack of recent studies, as well as limited reporting on co‐sensitization and clinical relevance. Large‐scale, multicenter epidemiological studies are needed in European countries to assess the effects of the 2019 EU legislation on public health. Additionally, many of the included studies failed to report on the clinical relevance of positive patch tests. Furthermore, the ability to quantify the precise rates of co‐sensitization between formaldehyde and its various releasers is limited. Future studies should address these issues to improve our understanding.

### 4.3 Conclusion

Contact allergy to formaldehyde and its releasers is a common and persistent problem among dermatitis patients worldwide. The significant disparity in sensitization rates suggests that regulatory interventions are effective public health measures.

## Author Contributions

**Kian Karimian:** conceptualization, investigation, writing – original draft, methodology, validation, writing – review and editing, project administration, supervision. **Daniel Isufi:** investigation, writing – review and editing, methodology, data curation, validation, resources. **Mikkel Bak Jensen:** software, supervision, formal analysis, writing – review and editing, visualization, validation, methodology, conceptualization. **Rebekka Søgaard:** conceptualization, methodology, writing – review and editing, validation, supervision. **Jeanne Duus Johansen:** conceptualization, validation, writing – review and editing, project administration, supervision. **Jakob Ferløv Baselius Schwensen:** conceptualization, validation, supervision, project administration, writing – review and editing.

## Funding

This work was supported by The Danish Environmental Protection Agency under the Ministry of Environment of Denmark.

## Conflicts of Interest

Mr. Karimian, Mr. Isufi, Dr. Jensen, Dr. Søgaard and Dr. Johansen have no conflicts of interest to declare. Dr. Schwensen has previously served as speaker and investigator for Galderma and Sanofi‐Aventis.

## Supporting information


**Table S1:** Search strings for databases.
**Table S2:** Appraisal tool for Cross‐Sectional Studies (AXIS) assessment of included studies.

## Data Availability

The data that support the findings of this study are available through the corresponding author.

## References

[1] A. C. De Groot , M. A. Flyvholm , G. Lensen , T. Menné , and P. J. Coenraads , “Formaldehyde‐Releasers: Relationship to Formaldehyde Contact Allergy. Contact Allergy to Formaldehyde and Inventory of Formaldehyde‐Releasers,” Contact Dermatitis 61, no. 2 (2009): 63–85, 10.1111/J.1600-0536.2009.01582.X. 19706047

[2] I. Hauksson , A. Pontén , M. Isaksson , H. Hamada , M. Engfeldt , and M. Bruze , “Formaldehyde in Cosmetics in Patch Tested Dermatitis Patients With and Without Contact Allergy to Formaldehyde,” Contact Dermatitis 74, no. 3 (2016): 145–151, 10.1111/COD.12493. 26696132

[3] T. Salthammer , “Data on Formaldehyde Sources, Formaldehyde Concentrations and Air Exchange Rates in European Housings,” Data in Brief 22 (2019): 400–435, 10.1016/J.DIB.2018.11.096. 30596137 PMC6309026

[4] I. M. Fasth , N. H. Ulrich , and J. D. Johansen , “Ten‐Year Trends in Contact Allergy to Formaldehyde and Formaldehyde‐Releasers,” Contact Dermatitis 79, no. 5 (2018): 263–269, 10.1111/COD.13052. 30079600

[5] W. Uter , S. M. Wilkinson , O. Aerts , et al., “Patch Test Results With the European Baseline Series, 2019/20—Joint European Results of the ESSCA and the EBS Working Groups of the ESCD, and the GEIDAC,” Contact Dermatitis 87, no. 4 (2022): 343–355, 10.1111/COD.14170. 35678309

[6] A. M. Giménez‐Arnau , G. Deza , A. Bauer , et al., “Contact Allergy to Preservatives: ESSCA* Results With the Baseline Series, 2009–2012,” Journal of the European Academy of Dermatology and Venereology 31, no. 4 (2017): 664–671, 10.1111/jdv.14063. 27896884

[7] T. Sanz‐Sánchez , P. M. García , J. F. Silvestre Salvador , et al., “Contact Allergy to Formaldehyde Releasers. Prospective Multicenter Study,” Contact Dermatitis 82, no. 3 (2020): 173–175, 10.1111/cod.13417. 31617596

[8] A. R. Atwater , A. J. Petty , B. Liu , et al., “Contact Dermatitis Associated With Preservatives: Retrospective Analysis of North American Contact Dermatitis Group Data, 1994 Through 2016,” Journal of the American Academy of Dermatology 84, no. 4 (2021): 965–976, 10.1016/j.jaad.2020.07.059. 33579596 PMC8087451

[9] J. G. DeKoven , E. M. Warshaw , K. A. Zug , et al., “North American Contact Dermatitis Group Patch Test Results: 2015‐2016,” Dermatitis 29, no. 6 (2018): 297–309, 10.1097/DER.0000000000000417. 30422882

[10] COMMISSION REGULATION (EU) , “Amending Annexes II, III and V to Regulation (EC) no 1223/2009 of the European Parliament and of the Council on Cosmetic Products,” Official Journal of the European Union 23, no. 5 (2019): 30–31.

[11] The European Parliament and the Council of the European Union Regulation (EC) , “The European Parliament and of the Council of 30 November 2009 on Cosmetic Products,” Official Journal of the European Union 342 (2009): 59.

[12] Commission Regulation (EU) , “Amending the Preamble of Annex V to Regulation (EC) no 1223/2009 of the European Parliament and of the Council on Cosmetic Products,” Official Journal of the European Union 2022 65 (2022): 3–4.

[13] D. Moher , A. Liberati , J. Tetzlaff , and D. G. Altman , “Preferred Reporting Items for Systematic Reviews and Meta‐Analyses: The PRISMA Statement,” BMJ (Online) 339, no. 7716 (2009): b2535, 10.1136/bmj.b2535. PMC2714657 19622551

[14] M. Ouzzani , H. Hammady , Z. Fedorowicz , and A. Elmagarmid , “Rayyan‐A Web and Mobile App for Systematic Reviews,” Systematic Reviews 5, no. 1 (2016): 1–10, 10.1186/S13643-016-0384-4. 27919275 PMC5139140

[15] M. J. Downes , M. L. Brennan , H. C. Williams , and R. S. Dean , “Development of a Critical Appraisal Tool to Assess the Quality of Cross‐Sectional Studies (AXIS),” BMJ Open 6, no. 12 (2016): e011458, 10.1136/BMJOPEN-2016-011458. PMC5168618 27932337

[16] F. J. Storrs , L. E. Rosenthal , R. M. Adams , et al., “Prevalence and Relevance of Allergic Reactions in Patients Patch Tested in North America—1984 to 1985,” Journal of the American Academy of Dermatology 20, no. 6 (1989): 1038–1045, 10.1016/S0190-9622(89)70129-8. 2754054

[17] D. L. Holness , J. R. Nethercott , R. M. Adams , et al., “Concomitant Positive Patch Test Results With Standard Screening Tray in North America 1985–1989,” Contact Dermatitis 32, no. 5 (1995): 289–292, 10.1111/j.1600-0536.1995.tb00783.x. 7634783

[18] S. Piaserico , F. Larese , G. P. Recchia , et al., “Allergic Contact Sensitivity in Elderly Patients,” Aging Clinical and Experimental Research 16, no. 3 (2004): 221–225, 10.1007/BF03327387. 15462465

[19] W. H. Wang , L. F. Li , X. Y. Lu , and J. Wang , “Cosmetic Dermatitis in Chinese Eczema Patients Patch Tested With a Modified European Standard Series of Allergens,” Contact Dermatitis 53, no. 6 (2005): 314–319, 10.1111/j.0105-1873.2005.00717.x. 16364117

[20] M. N. Kashani , F. Gorouhi , F. Behnia , M. J. Nazemi , Y. Dowlati , and A. Firooz , “Allergic Contact Dermatitis in Iran,” Contact Dermatitis 52, no. 3 (2005): 154–158, 10.1111/J.0105-1873.2005.00545.X. 15811031

[21] D. A. Wetter , M. D. P. Davis , J. A. Yiannias , et al., “Patch Test Results From the Mayo Clinic Contact Dermatitis Group, 1998‐2000,” Journal of the American Academy of Dermatology 53, no. 3 (2005): 416–421, 10.1016/j.jaad.2005.04.077. 16112346

[22] A. Boyvat , A. Akyol , and E. Gürgey , “Contact Sensitivity to Preservatives in Turkey,” Contact Dermatitis 52, no. 6 (2005): 329–332, 10.1111/j.0105-1873.2005.00607.x. 15932584

[23] A. Lazarov , “European Standard Series Patch Test Results From a Contact Dermatitis Clinic in Israel During the 7‐Year Period From 1998 to 2004,” Contact Dermatitis 55, no. 2 (2006): 73–76, 10.1111/j.0105-1873.2006.00875.x. 16930229

[24] M. Hogeling and M. Pratt , “Allergic Contact Dermatitis in Children: The Ottawa Hospital Patch‐Testing Clinic Experience, 1996 to 2006,” Dermatitis 19, no. 2 (2008): 86–89, 10.2310/6620.2008.07099. 18413109

[25] D. A. Wetter , J. A. Yiannias , A. V. Prakash , M. D. P. Davis , S. A. Farmer , and R. A. El‐Azhary , “Results of Patch Testing to Personal Care Product Allergens in a Standard Series and a Supplemental Cosmetic Series: An Analysis of 945 Patients From the Mayo Clinic Contact Dermatitis Group, 2000‐2007,” Journal of the American Academy of Dermatology 63, no. 5 (2010): 789–798, 10.1016/j.jaad.2009.11.033. 20643495

[26] K. D. Bilcha , A. Ayele , D. Shibeshi , and C. Lovell , “Patch Testing and Contact Allergens in Ethiopia – Results of 514 Contact Dermatitis Patients Using the European Baseline Series,” Contact Dermatitis 63, no. 3 (2010): 140–145, 10.1111/J.1600-0536.2010.01740.X. 20690936

[27] S. E. Jacob , A. Yang , E. Herro , and C. Zhang , “Contact Allergens in a Pediatric Population: Association With Atopic Dermatitis and Comparison With Other North American Referral Centers,” Journal of Clinical and Aesthetic Dermatology 3, no. 10 (2010): 1–7. PMC2958194 20967193

[28] M. T. Bordel‐Gómez , A. Miranda‐Romero , and J. Castrodeza‐Sanz , “Epidemiology of Contact Dermatitis: Prevalence of Sensitization to Different Allergens and Associated Factors,” Actas Dermo‐Sifiliográficas (English Edition) 101, no. 1 (2010): 59–75, 10.1016/S1578-2190(10)70581-3. 20109394

[29] S. Cheng , M. Cao , Y. Zhang , et al., “Time Trends of Contact Allergy to a Modified European Baseline Series in Beijing Between 2001 and 2006,” Contact Dermatitis 65, no. 1 (2011): 22–27, 10.1111/J.1600-0536.2011.01897.X. 21599703

[30] W. Boonchai , R. Desomchoke , and P. Iamtharachai , “Trend of Contact Allergy to Cosmetic Ingredients in Thais Over a Period of 10 Years,” Contact Dermatitis 65, no. 6 (2011): 311–316, 10.1111/j.1600-0536.2011.01978.x. 22077433

[31] R. Yin , X. Y. Huang , X. F. Zhou , and F. Hao , “A Retrospective Study of Patch Tests in Chongqing, China From 2004 to 2009,” Contact Dermatitis 65, no. 1 (2011): 28–33, 10.1111/J.1600-0536.2010.01854.X. 21309787

[32] A. Machovcova , “The Frequency of Contact Allergy in Children and Adolescents in The Czech Republic,” Acta Dermatovenerologica Croatica 20, no. 2 (2012): 75–79. 22726278

[33] S. S. Lee , D. K. Hong , N. J. Jeong , et al., “Multicenter Study of Preservative Sensitivity in Patients With Suspected Cosmetic Contact Dermatitis in Korea,” Journal of Dermatology 39, no. 8 (2012): 677–681, 10.1111/j.1346-8138.2012.01551.x. 22548403

[34] E. M. Warshaw , D. V. Belsito , J. S. Taylor , et al., “North American Contact Dermatitis Group Patch Test Results: 2009 to 2010,” Dermatitis 24, no. 2 (2013): 50–59, 10.1097/DER.0b013e3182819c51. 23474444

[35] E. Ç. Özel and K. Özyurt , “Patch Test Results in Patients With Allergic Contact Dermatitis in Yozgat,” Turkderm Deri Hastaliklari Ve Frengi Arsivi 47, no. 3 (2013): 161–165, 10.4274/TURKDERM.81905.

[36] A. B. Wentworth , J. A. Yiannias , and J. H. Keeling , “Trends in Patch‐Test Results and Allergen Changes in the Standard Series: A Mayo Clinic 5‐Year Retrospective Review,” Journal of the American Academy of Dermatology 70, no. 2 (2014): 269–275, 10.1016/j.jaad.2013.09.047. 24268786

[37] M. Pesonen , R. Jolanki , F. Larese Filon , et al., “Patch Test Results of the European Baseline Series Among Patients With Occupational Contact Dermatitis Across Europe – Analyses of the European Surveillance System on Contact Allergy Network, 2002–2010,” Contact Dermatitis 72, no. 3 (2015): 154–163, 10.1111/COD.12333. 25639629

[38] E. M. Warshaw , H. I. Maibach , J. S. Taylor , et al., “North American Contact Dermatitis Group Patch Test Results: 2011‐2012,” Dermatitis 26, no. 1 (2015): 49–59, 10.1097/DER.0000000000000097. 25581671

[39] A. Prodi , F. Rui , A. Belloni Fortina , M. T. Corradin , and F. F. Larese , “Sensitization to Formaldehyde in Northeastern Italy, 1996 to 2012,” Dermatitis 27, no. 1 (2016): 21–25, 10.1097/DER.0000000000000158. 26756512

[40] D. S. Yu , H. J. Kim , Y. G. Park , J. M. Bae , J. W. Kim , and Y. B. Lee , “Patch‐Test Results Using Korean Standard Series: A 5‐Year Retrospective Review,” Journal of Dermatological Treatment 28, no. 3 (2017): 258–262, 10.1080/09546634.2016.1219015. 27469077

[41] K. K. Veverka , M. R. Hall , J. A. Yiannias , et al., “Trends in Patch Testing With the Mayo Clinic Standard Series, 2011‐2015,” Dermatitis 29, no. 6 (2018): 310–315, 10.1097/DER.0000000000000411. 30422883

[42] S. Rastogi , K. R. Patel , V. Singam , and J. I. Silverberg , “Allergic Contact Dermatitis to Personal Care Products and Topical Medications in Adults With Atopic Dermatitis,” Journal of the American Academy of Dermatology 79, no. 6 (2018): 1028–1033.e6, 10.1016/j.jaad.2018.07.017. 30053491

[43] A. B. Simonsen , M. H. Foss‐Skiftesvik , J. P. Thyssen , et al., “Contact Allergy in Danish Children: Current Trends,” Contact Dermatitis 79, no. 5 (2018): 295–302, 10.1111/cod.13079. 30094861

[44] T. Garg , S. Agarwal , R. Chander , A. Singh , and P. Yadav , “Patch Testing in Patients With Suspected Cosmetic Dermatitis: A Retrospective Study,” Journal of Cosmetic Dermatology 17, no. 1 (2018): 95–100, 10.1111/jocd.12359. 28568892

[45] Y. K. Huang , Y. H. Wu , P. H. Lu , and M. E. Tu , “Contact Allergy to Preservatives in Taiwan Between 1996 and 2015,” Dermatologica Sinica 37, no. 3 (2019): 123, 10.4103/ds.ds_21_18.

[46] M. Tunca , E. Çaliskan , and A. Yürekli , “Frequent Contact Allergens in Ankara/Turkey: A Retrospective Study of Patch Test Results,” Turkderm Turkish Archives of Dermatology and Venereology 53, no. 2 (2019): 49–52, 10.4274/turkderm.galenos.2018.68335.

[47] P. U. Ibekwe and Z. S. Bagudu , “Patch Test Results Obtained With the European Baseline Series at a Diagnostics Centre in Abuja, Nigeria,” Contact Dermatitis 81, no. 2 (2019): 154–155, 10.1111/cod.13274. 30903665

[48] A. Tagka , A. Stratigos , G. I. Lambrou , E. Nicolaidou , A. Katsarou , and A. Chatziioannou , “Prevalence of Contact Dermatitis in the Greek Population: A Retrospective Observational Study,” Contact Dermatitis 81, no. 6 (2019): 460–462, 10.1111/cod.13364. 31347179

[49] T. Sukakul , P. Chaweekulrat , P. Limphoka , and W. Boonchai , “Changing Trends of Contact Allergens in Thailand: A 12‐Year Retrospective Study,” Contact Dermatitis 81, no. 2 (2019): 124–129, 10.1111/cod.13289. 30977136

[50] I. Tam , P. C. Schalock , E. González , and J. Yu , “Patch Testing Results From the Massachusetts General Hospital Contact Dermatitis Clinic, 2007‐2016,” Dermatitis 31, no. 3 (2020): 202–208, 10.1097/DER.0000000000000593. 32209868

[51] E. A. Silva , M. R. M. Bosco , R. R. Lozano , A. C. P. Latini , and S. V. N. B. de , “High Rate of Sensitization to Kathon CG, Detected by Patch Tests in Patients With Suspected Allergic Contact Dermatitis,” Anais Brasileiros de Dermatologia 95, no. 2 (2020): 194–199, 10.1016/j.abd.2019.09.026. 32156503 PMC7175102

[52] O. Qin , Y. Cheng , W. Hu , et al., “Patch Test in Chinese in Shanghai With Cosmetic Allergy to Cosmetic Series and Products,” Journal of Cosmetic Dermatology 19, no. 8 (2020): 2086–2092, 10.1111/jocd.13249. 31820565

[53] R. W. Collis , G. M. Morris , D. M. Sheinbein , and C. C. Coughlin , “Expanded Series and Personalized Patch Tests for Children: A Retrospective Cohort Study,” Dermatitis 31, no. 2 (2020): 144–146, 10.1097/DER.0000000000000506. 31441780

[54] A. Dugonik , B. Dugonik , V. Podgorelec , and L. Brezočnik , “Associated Positive Patch Test Reactions to Standard Contact Allergens: 10‐Year Data From the Slovenian E‐Surveillance System,” Contact Dermatitis 85, no. 1 (2021): 17–25, 10.1111/cod.13767. 33368304

[55] E. Yücel and D. Özçeker , “Contact Allergen Sensitivity in Children With Contact Dermatitis,” Turkish Archives of Pediatrics 56, no. 1 (2021): 51–56, 10.14744/TURKPEDIATRIARS.2020.79577. 34013230 PMC8114594

[56] A. Boyvat and Y. I. Kalay , “Patch Test Results of the European Baseline Series Among 1309 Patients in Turkey Between 2013 and 2019,” Contact Dermatitis 84, no. 1 (2021): 15–23, 10.1111/COD.13653. 32618364

[57] C. Wee , C. H. Tan , X. Zhao , Y. W. Yew , and A. Goon , “Pattern of Contact Sensitization in Patients With and Without Atopic Dermatitis in an Asian Dermatology Center,” Contact Dermatitis 86, no. 5 (2022): 398–403, 10.1111/COD.14068. 35133669

[58] E. Noë , S. Huygens , M. A. Morren , M. Garmyn , A. Goossens , and L. Gilissen , “Contact Allergy in a Paediatric Population Observed in a Tertiary Referral Centre in Belgium,” Contact Dermatitis 86, no. 1 (2022): 3–8, 10.1111/cod.13975. 34537955

[59] W. Boonchai , C. Pruksaeakanan , S. Wongdama , M. Bunyavaree , T. Kumpangsin , and C. Chaiyabutr , “Trends in Formaldehyde and Formaldehyde‐Releaser Contact Allergies as Compared With Market Exposure in Thailand,” Contact Dermatitis 88, no. 1 (2023): 18–26, 10.1111/cod.14190. 35838492

[60] D. Slodownik , J. Bar , and D. Daniely , “Trends in Contact Sensitization, Results, and Implications From a Contact Dermatitis Clinic in Israel,” Contact Dermatitis 90, no. 6 (2024): 556–565, 10.1111/cod.14524. 38368629

[61] L. Piapan , A. Belloni Fortina , E. Giulioni , and F. F. Larese , “Sensitization to Ethylenediamine Dihydrochloride in Patients With Contact Dermatitis in Northeastern Italy From 1996 to 2021,” Contact Dermatitis 90, no. 3 (2024): 253–261, 10.1111/cod.14454. 38038148

[62] N. Belhadj , A. Brahem , N. B. Chabbah , et al., “Predictive Factors of Polysensitization to Contact Allergens: Study Conducted at the Dermato‐Allergology Unit of the Farhat Hached University Hospital in Sousse, Tunisia,” Pan African Medical Journal 48, no. 40 (2024): 1–14, 10.11604/PAMJ.2024.48.40.35295. 39280828 PMC11399470

[63] R. Søgaard , C. Kursawe Larsen , J. D. Johansen , and J. F. B. Schwensen , “Trends in Contact Allergy to Preservatives From 2014 to 2023: Benzisothiazolinone on the Rise,” Contact Dermatitis 93, no. 3 (2025): 214–223, 10.1111/COD.14818. 40443262 PMC12318914

[64] R. M. Adams , H. I. Maibach , and W. E. Clendenning , “A Five‐Year Study of Cosmetic Reactions,” Journal of the American Academy of Dermatology 13, no. 6 (1985): 1062–1069, 10.1016/S0190-9622(85)70258-7. 4078100

[65] J. Marks , D. V. Belsito , V. A. DeLeo , et al., “North American Contact Dermatitis Group Patch Test Results for the Detection of Delayed‐Type Hypersensitivity to Topical Allergens,” Journal of the American Academy of Dermatology 38, no. 6 I (1998): 911–918, 10.1016/S0190-9622(98)70587-0. 9631997

[66] J. G. Marks , D. V. Belsito , V. A. DeLeo , et al., “North American Contact Dermatitis Group Standard Tray Patch Test Results (1992 to 1994),” American Journal of Contact Dermatitis 6, no. 3 (1995): 160–165, 10.1016/1046-199X(95)90122-1.

[67] A. Schnuch , J. Geier , W. Uter , and P. J. Frosch , “Patch Testing With Preservatives, Antimicrobials and Industrial Biocides. Results From a Multicentre Study,” British Journal of Dermatology 138, no. 3 (1998): 467–476, 10.1046/j.1365-2133.1998.02126.x. 9580801

[68] R. L. Rietschel , C. G. T. Mathias , J. F. Fowler , et al., “Relationship of Occupation to Contact Dermatitis: Evaluation in Patients Tested From 1998 to 2000,” American Journal of Contact Dermatitis 13, no. 4 (2002): 170–176, 10.1053/ajcd.2002.36635. 12478531

[69] E. Akasya‐Hillenbrand and E. Özkaya‐Bayazit , “Patch Test Results in 542 Patients With Suspected Contact Dermatitis in Turkey,” Contact Dermatitis 46, no. 1 (2002): 17–23, 10.1034/j.1600-0536.2002.460104.x. 11918582

[70] J. Brasch , A. Schnuch , and W. Uter , “Patch‐Test Reaction Patterns in Patients With a Predisposition to Atopic Dermatitis,” Contact Dermatitis 49, no. 4 (2003): 197–201, 10.1111/j.0105-1873.2003.0227.x. 14996068

[71] J. E. R. Britton , S. M. Wilkinson , J. S. C. English , et al., “The British Standard Series of Contact Dermatitis Allergens: Validation in Clinical Practice and Value for Clinical Governance,” British Journal of Dermatology 148, no. 2 (2003): 259–264, 10.1046/j.1365-2133.2003.05170.x. 12588377

[72] W. Uter , J. Hegewald , W. Aberer , et al., “The European Standard Series in 9 European Countries, 2002/2003 – First Results of the European Surveillance System on Contact Allergies,” Contact Dermatitis 53, no. 3 (2005): 136–145, 10.1111/J.0105-1873.2005.00673.X. 16128752

[73] A. Machovcova , E. Dastychova , D. Kostalova , et al., “Common Contact Sensitizers in The Czech Republic. Patch Test Results in 12,058 Patients With Suspected Contact Dermatitis*,” Contact Dermatitis 53, no. 3 (2005): 162–166, 10.1111/J.0105-1873.2005.00676.X. 16128756

[74] L. F. Li , G. Liu , and J. Wang , “Patch Test in Chinese Patients With Cosmetic Allergic Contact Dermatitis to Common Cosmetic Allergens From a European Cosmetic Series,” Contact Dermatitis 57, no. 1 (2007): 50–54, 10.1111/j.1600-0536.2007.01073.x. 17577360

[75] K. A. Zug , D. McGinley‐Smith , E. M. Warshaw , et al., “Contact Allergy in Children Referred for Patch Testing: North American Contact Dermatitis Group Data, 2001‐2004,” Archives of Dermatology 144, no. 10 (2008): 1329–1336, 10.1001/ARCHDERM.144.10.1329. 18936397

[76] K. A. Zug , E. M. Warshaw , J. F. Fowler , et al., “Patch‐Test Results of the North American Contact Dermatitis Group 2005‐2006,” Dermatitis 20, no. 3 (2009): 149–160, 10.2310/6620.2009.08097. 19470301

[77] J. P. Thyssen , K. Engkilde , M. D. Lundov , B. C. Carlsen , T. Menné , and J. D. Johansen , “Temporal Trends of Preservative Allergy in Denmark (1985‐2008),” Contact Dermatitis 62, no. 2 (2010): 102–108, 10.1111/j.1600-0536.2009.01668.x. 20136893

[78] A. Beliauskiene , S. Valiukeviciene , W. Uter , and A. Schnuch , “The European Baseline Series in Lithuania: Results of Patch Testing in Consecutive Adult Patients,” Journal of the European Academy of Dermatology and Venereology 25, no. 1 (2011): 59–63, 10.1111/J.1468-3083.2010.03688.X. 20456555

[79] E. M. Herro , C. Matiz , K. Sullivan , C. Hamann , and S. E. Jacob , “Frequency of Contact Allergens in Pediatric Patients With Atopic Dermatitis,” Journal of Clinical and Aesthetic Dermatology 4, no. 11 (2011): 39–41. PMC3225137 22125658

[80] M. Moustafa , C. R. Holden , P. Athavale , M. J. Cork , A. G. Messenger , and D. J. Gawkrodger , “Patch Testing Is a Useful Investigation in Children With Eczema,” Contact Dermatitis 65, no. 4 (2011): 208–212, 10.1111/j.1600-0536.2011.01900.x. 21504435

[81] L. Landeck , P. Schalock , L. Baden , and E. González , “Contact Sensitization Pattern in 172 Atopic Subjects,” International Journal of Dermatology 50, no. 7 (2011): 806–810, 10.1111/J.1365-4632.2010.04754.X. 21699515

[82] N. Latorre , L. Borrego , V. Fernández‐Redondo , et al., “Patch Testing With Formaldehyde and Formaldehyde‐Releasers: Multicentre Study in Spain (2005‐2009),” Contact Dermatitis 65, no. 5 (2011): 286–292, 10.1111/j.1600-0536.2011.01953.x. 21767276

[83] J. P. Thyssen , J. D. Johansen , A. Linneberg , T. Menné , and K. Engkilde , “The Association Between Contact Sensitization and Atopic Disease by Linkage of a Clinical Database and a Nationwide Patient Registry,” Allergy 67, no. 9 (2012): 1157–1164, 10.1111/J.1398-9995.2012.02863.X. 22765654

[84] M. A. Bernier , A. I. Bernal‐Ruiz , F. Rivas‐Ruiz , M. T. Fernández‐Morano , and M. De Troya‐Martín , “Contact Sensitization to Allergens in the Spanish Standard Series at Hospital Costa Del Sol in Marbella, Spain: A Retrospective Study (2005‐2010),” Actas Dermo‐Sifiliográficas 103, no. 3 (2012): 223–228, 10.1016/j.ad.2011.07.010. 21963222

[85] E. M. Warshaw , S. I. Raju , J. F. Fowler , et al., “Positive Patch Test Reactions in Older Individuals: Retrospective Analysis From the North American Contact Dermatitis Group, 1994‐2008,” Journal of the American Academy of Dermatology 66, no. 2 (2012): 229–240, 10.1016/j.jaad.2010.12.022. 21596455

[86] A. F. Fransway , K. A. Zug , D. V. Belsito , et al., “North American Contact Dermatitis Group Patch Test Results for 2007‐2008,” Dermatitis 24, no. 1 (2013): 10–21, 10.1097/DER.0b013e318277ca50. 23340394

[87] A. B. Simonsen , M. Deleuran , C. G. Mortz , J. D. Johansen , and M. Sommerlund , “Allergic Contact Dermatitis in Danish Children Referred for Patch Testing – A Nationwide Multicentre Study,” Contact Dermatitis 70, no. 2 (2014): 104–111, 10.1111/COD.12129. 24102181

[88] L. Malinauskiene , M. Bruze , and M. Isaksson , “Patch Testing With the Swedish Baseline Series in Two Countries,” Journal of Clinical & Experimental Dermatology Research 6, no. 5 (2015): 1–6, 10.4172/2155-9554.10000299.

[89] K. K. B. Clemmensen , S. F. Thomsen , G. B. E. Jemec , and T. Agner , “Pattern of Contact Sensitization in Patients With and Without Atopic Dermatitis in a Hospital‐Based Clinical Database,” Contact Dermatitis 71, no. 2 (2014): 75–81, 10.1111/cod.12229. 24697272

[90] R. Toholka , Y. S. Wang , B. Tate , et al., “The First Australian Baseline Series: Recommendations for Patch Testing in Suspected Contact Dermatitis,” Australasian Journal of Dermatology 56, no. 2 (2015): 107–115, 10.1111/AJD.12186. 25196101

[91] A. Dinkloh , M. Worm , J. Geier , A. Schnuch , and A. Wollenberg , “Contact Sensitization in Patients With Suspected Cosmetic Intolerance: Results of the IVDK 2006‐2011,” Journal of the European Academy of Dermatology and Venereology 29, no. 6 (2015): 1071–1081, 10.1111/jdv.12750. 25288472

[92] K. Linauskienė , L. Malinauskienė , and A. Blažienė , “Time Trends of Contact Allergy to the European Baseline Series in Lithuania,” Contact Dermatitis 76, no. 6 (2017): 350–356, 10.1111/COD.12726. 27917496

[93] E. M. Warshaw , M. C. Goodier , J. G. Dekoven , et al., “Contact Dermatitis Associated With Skin Cleansers: Retrospective Analysis of North American Contact Dermatitis Group Data 2000‐2014,” Dermatitis 29, no. 1 (2018): 32–42, 10.1097/DER.0000000000000330. 29256904

[94] A. B. Simonsen , J. D. Johansen , M. Deleuran , C. G. Mortz , L. Skov , and M. Sommerlund , “Children With Atopic Dermatitis May Have Unacknowledged Contact Allergies Contributing to Their Skin Symptoms,” Journal of the European Academy of Dermatology and Venereology 32, no. 3 (2018): 428–436, 10.1111/jdv.14737. 29222945

[95] F. Peng , R. A. Schwartz , Z. Chen , and J. Z. Zhang , “High Prevalence of Contact Hypersensitivity to Metals and Preservatives in Chinese Patients With Atopic Dermatitis,” Chinese Medical Journal 132, no. 23 (2019): 2881–2882, 10.1097/CM9.0000000000000526. 31856061 PMC6940085

[96] Y. Teo , J. P. McFadden , I. R. White , M. Lynch , and P. Banerjee , “Allergic Contact Dermatitis in Atopic Individuals: Results of a 30‐Year Retrospective Study,” Contact Dermatitis 81, no. 6 (2019): 409–416, 10.1111/COD.13363. 31347185

[97] C. Felmingham , R. Davenport , H. Bala , A. Palmer , and R. Nixon , “Allergic Contact Dermatitis in Children and Proposal for an Australian Paediatric Baseline Series,” Australasian Journal of Dermatology 61, no. 1 (2020): 33–38, 10.1111/AJD.13169. 31630402

[98] C. P. Hernández‐Fernández , P. Mercader‐García , J. F. Silvestre Salvador , et al., “Candidate Allergens for Inclusion in the Spanish Standard Series Based on Data From the Spanish Contact Dermatitis Registry,” Actas Dermo‐Sifiliográficas 112, no. 9 (2021): 798–805, 10.1016/j.ad.2021.05.005. 34029518

[99] K. Dear , H. Bala , A. Palmer , and R. L. Nixon , “How Good Is the Australian Baseline Series at Detecting Allergic Contact Dermatitis?,” Australasian Journal of Dermatology 62, no. 1 (2021): 51–56, 10.1111/ajd.13456. 32914863

[100] R. Koca , E. Kocaturk , E. Savk , et al., “Patch Test Results to European Baseline Series in Turkey: A Prospective and Multicenter Study,” Dermatitis 32, no. 6 (2021): 397–405, 10.1097/DER.0000000000000631. 33731647

[101] W. Uter , A. Zetzmann , R. Ofenloch , et al., “Prevalence of Contact Allergies in the Population Compared to a Tertiary Referral Patch Test Clinic in Jena/Germany,” Contact Dermatitis 85, no. 5 (2021): 563–571, 10.1111/COD.13923. 34184275

[102] J. I. Silverberg , A. Hou , E. M. Warshaw , et al., “Prevalence and Trend of Allergen Sensitization in Adults and Children With Atopic Dermatitis Referred for Patch Testing, North American Contact Dermatitis Group Data, 2001‐2016,” Journal of Allergy and Clinical Immunology: In Practice 9, no. 7 (2021): 2853–2866, 10.1016/j.jaip.2021.03.028. 33781959

[103] W. Boonchai , C. Chaiyabutr , N. Charoenpipatsin , and T. Sukakul , “Pediatric Contact Allergy: A Comparative Study With Adults,” Contact Dermatitis 84, no. 1 (2021): 34–40, 10.1111/cod.13672. 32696982

[104] A. Ito , K. Suzuki , K. Matsunaga , et al., “Patch Testing With the Japanese Baseline Series 2015: A 4‐Year Experience,” Contact Dermatitis 86, no. 3 (2022): 189–195, 10.1111/cod.14027. 34921568

[105] A. Bauer , M. Pesonen , R. Brans , et al., “Occupational Contact Allergy: The European Perspective–Analysis of Patch Test Data From ESSCA Between 2011 and 2020,” Contact Dermatitis 88, no. 4 (2023): 263–274, 10.1111/cod.14280. 36694979

[106] P. Puangpet , N. Boonpuen , K. Saipornchai , P. Poompakdeepan , R. Suchaoin , and J. McFadden , “Patch Testing of Thai Children With Eczema,” Siriraj Medical Journal 75, no. 2 (2023): 70–75, 10.33192/smj.v75i2.260740.

[107] J. C. Carvalho , I. A. Coutinho , C. Loureiro , A. C. Cordeiro , L. Ramos , and M. Gonçalo , “Contact Sensitization in Pediatric Patients With Atopic Dermatitis: A Purpose for a New Patch Testing Series for the Portuguese Population,” European Annals of Allergy and Clinical Immunology 56, no. 1 (2024): 9, 10.23822/EurAnnACI.1764-1489.258. 35686363

[108] S. L. Husajn , “Contact Dermatitis in the West of Scotland,” Contact Dermatitis 3, no. 6 (1977): 327–332, 10.1111/j.1600-0536.1977.tb03697.x. 580079

[109] A. C. de Groot , D. H. Liem , J. P. Nater , and W. G. van Ketel , “Patch Tests With Fragrance Materials and Preservatives,” Contact Dermatitis 12, no. 2 (1985): 87–92, 10.1111/j.1600-0536.1985.tb01059.x. 3987262

[110] C. M. Perret and R. Happle , “Contact Sensitivity to Diazolidinyl Urea (Germall II),” Archives of Dermatological Research 281, no. 1 (1989): 57–59, 10.1007/BF00424274. 2730144

[111] M.‐C. Jacobs , I. R. White , R. J. G. Rycroft , and N. Taub , “Patch Testing With Preservatives at St John's From 1982 to 1993,” Contact Dermatitis 33, no. 4 (1995): 247–254, 10.1111/j.1600-0536.1995.tb00476.x. 8654076

[112] M. Kiec‐Swierczynska , “Occupational Allergic Contact Dermatitis in Lodz: 1990‐1994,” Occupational Medicine (Chic Ill) 46, no. 3 (1996): 205–208, 10.1093/occmed/46.3.205. 8695772

[113] N. Henrik Nielsen , A. Linneberg , T. Menné , et al., “Allergic Contact Sensitization in an Adult Danish Population: Two Cross‐Sectional Surveys Eight Years Apart (The Copenhagen Allergy Study),” Acta Dermato‐Venereologica 81, no. 1 (2001): 31–34, 10.1080/000155501750208155. 11411911

[114] W. Uter and P. J. Frosch , “Contact Allergy From DMDM Hydantoin, 1994‐2000,” Contact Dermatitis 47, no. 1 (2002): 57–58, 10.1034/j.1600-0536.2002.470119.x. 12225423

[115] A. Lindberg , M. Tammela , Å. Boström , et al., “Are Adverse Skin Reactions to Cosmetics Underestimated in the Clinical Assessment of Contact Dermatitis? A Prospective Study Among 1075 Patients Attending Swedish Patch Test Clinics,” Acta Dermato‐Venereologica 84, no. 4 (2004): 291–295, 10.1080/00015550410025921. 15339074

[116] D. P. Bruynzeel , T. L. Diepgen , K. E. Andersen , et al., “Monitoring the European Standard Series in 10 Centres 1996–2000,” Contact Dermatitis 53, no. 3 (2005): 146–149, 10.1111/J.0105-1873.2005.00541.X. 16128753

[117] T. Hasan , T. Rantanen , K. Alanko , et al., “Patch Test Reactions to Cosmetic Allergens in 1995‐1997 and 2000‐2002 in Finland ‐ A Multicentre Study,” Contact Dermatitis 53, no. 1 (2005): 40–45, 10.1111/j.0105-1873.2005.00630.x. 15982231

[118] M. Kieć‐Swierczyńska , B. Krecisz , and D. Swierczyńska‐Machura , “Contact Allergy to Preservatives Contained in Cosmetics,” Medycyna Pracy 57, no. 3 (2006): 245–249. 17125030

[119] S. Racheva , “Etiology of Common Contact Dermatitis,” Journal of IMAB 12, no. 1 (2006): 22–25.

[120] C. T. Jong , B. N. Statham , C. M. Green , et al., “Contact Sensitivity to Preservatives in the UK, 2004‐2005: Results of Multicentre Study,” Contact Dermatitis 57, no. 3 (2007): 165–168, 10.1111/j.1600-0536.2007.01181.x. 17680865

[121] A. Schnuch , W. Uter , H. Lessmann , and J. Geier , “Contact Allergy to Preservatives. Results of the Information Network of Departments of Dermatology (IVDK) 1996 to 2007,” Allergo Journal 17, no. 8 (2008): 631–638, 10.1007/bf03361953.

[122] I. Hauksson , A. Pontén , B. Gruvberger , M. Isaksson , and M. Bruze , “Routine Diagnostic Patch‐Testing With Formaldehyde 2.0% (0.6 Mg/cm^2^) May Be an Advantage Compared to 1.0%,” Acta Dermato‐Venereologica 90, no. 5 (2010): 480–484, 10.2340/00015555-0925. 20814622

[123] P. Helsing , P. Gjersvik , H. JØi , et al., “Variability in Patch Test Reactions ‐ First Report From the Norwegian Patch Test Registry,” Contact Dermatitis 62, no. 5 (2010): 309–313, 10.1111/j.1600-0536.2010.01711.x. 20536479

[124] A. C. De Groot , J. Blok , and P. J. Coenraads , “Relationship Between Formaldehyde and Quaternium‐15 Contact Allergy. Influence of Strength of Patch Test Reactions,” Contact Dermatitis 63, no. 4 (2010): 187–191, 10.1111/j.1600-0536.2010.01712.x. 20573164

[125] M. Klimańska , M. Zmudzińska , D. Jenerowicz , and M. Czarnecka‐Operacz , “The Importance of Exposure to Contact Allergens in Patients With Allergic Contact Dermatitis,” Postepy Dermatologii I Alergologii 28, no. 3 (2011): 203–211.

[126] A. Schnuch , H. Lessmann , J. Geier , and W. Uter , “Contact Allergy to Preservatives. Analysis of IVDK Data 1996‐2009,” British Journal of Dermatology 164, no. 6 (2011): 1316–1325, 10.1111/j.1365-2133.2011.10253.x. 21332463

[127] M. Kiec‐Swierczynska , B. Krȩcisz , D. Chomiczewska , and W. Sobala , “Trends in Allergy to the 10 Most Frequent Contact Allergens in Patients Examined at the Nofer Institute, Lodz, Poland in 1996‐2009,” Postepy Dermatologii I Alergologii 29, no. 1 (2012): 19–24.

[128] W. Uter , W. Aberer , J. C. Armario‐Hita , et al., “Current Patch Test Results With the European Baseline Series and Extensions to It From the “European Surveillance System on Contact Allergy” Network, 2007‐2008,” Contact Dermatitis 67, no. 1 (2012): 9–19, 10.1111/J.1600-0536.2012.02070.X. 22500724

[129] M. Isaksson , J. Bråred‐Christensson , M. Engfeldt , et al., “Patch Testing With Formaldehyde 2.0% in Parallel With 1.0% by the Swedish Contact Dermatitis Research Group,” Acta Dermato‐Venereologica 94, no. 4 (2014): 408–410, 10.2340/00015555-1748. 24337098

[130] B. Kręcisz , D. Chomiczewska‐Skóra , and M. Kieć‐Świerczyńska , “Preservatives as Important Etiologic Factors of Allergic Contact Dermatitis,” Medycyna Pracy 66, no. 3 (2015): 327–332, 10.13075/mp.5893.00176. 26325045

[131] M. Hervella‐Garcés , J. García‐Gavín , and J. F. Silvestre‐Salvador , “The Spanish Standard Patch Test Series: 2016 Update by the Spanish Contact Dermatitis and Skin Allergy Research Group (GEIDAC),” Actas Dermo‐Sifiliográficas 107, no. 7 (2016): 559–566, 10.1016/j.adengl.2016.06.002. 27262363

[132] M. Lagrelius , C. F. Wahlgren , M. Matura , I. Kull , and C. Lidén , “High Prevalence of Contact Allergy in Adolescence: Results From the Population‐Based BAMSE Birth Cohort,” Contact Dermatitis 74, no. 1 (2016): 44–51, 10.1111/cod.12492. 26538115

[133] A. Pontén , M. Bruze , M. Engfeldt , I. Hauksson , and M. Isaksson , “Concomitant Contact Allergies to Formaldehyde, Methylchloroisothiazolinone/Methylisothiazolinone, Methylisothiazolinone, and Fragrance Mixes I and II,” Contact Dermatitis 75, no. 5 (2016): 285–289, 10.1111/cod.12598. 27145058

[134] E. Kasumagic‐Halilovic and N. Ovcina‐Kurtovic , “Analysis of Epicutaneous Patch Test Results in Patients With Contact Dermatitis,” Medical Archives 72, no. 4 (2018): 276–279, 10.5455/medarh.2018.72.276-279. 30514994 PMC6195022

[135] K. Aalto‐Korte and M. Pesonen , “Patterns of Positive Patch Test Reactions to Formaldehyde and Formaldehyde Releasers at the Finnish Institute of Occupational Health From 2007 to 2020,” Contact Dermatitis 85, no. 4 (2021): 429–434, 10.1111/cod.13876. 33934369

[136] H. Whitehouse , W. Uter , J. Geier , et al., “Formaldehyde 2% Is Not a Useful Means of Detecting Allergy to Formaldehyde Releasers—Results of the ESSCA Network, 2015‐2018,” Contact Dermatitis 84, no. 2 (2021): 95–102, 10.1111/cod.13691. 32876992

[137] D. Andernord , M. Bruze , I. L. Bryngelsson , et al., “Contact Allergy to Haptens in the Swedish Baseline Series: Results From the Swedish Patch Test Register (2010 to 2017),” Contact Dermatitis 86, no. 3 (2022): 175–188, 10.1111/COD.13996. 34704261

[138] M. Bizjak , K. Adamič , N. Bajrovič , et al., “Patch Testing With the European Baseline Series and 10 Added Allergens: Single‐Centre Study of 748 Patients,” Contact Dermatitis 87, no. 5 (2022): 439–446, 10.1111/cod.14178. 35736503 PMC9796124

[139] F. J. Navarro‐Triviño , L. Borrego , J. F. Silvestre‐Salvador , et al., “[Translated Article] Standard and Expanded Series Patch Testing Update by the Spanish Contact Dermatitis and Skin Allergy Research Group (GEIDAC),” Actas Dermo‐Sifiliográficas 115, no. 7 (2024): T712–T721, 10.1016/j.ad.2024.05.018. 38823769

[140] M. Erkoç , G. Özden , L. Çevirme , R. S. Cansunar , H. Basır , and S. Dik , “Outcomes of the European Baseline Series Patch Test in the Geriatric Population,” Allergol Immunopathol (Madr) 53, no. 4 (2025): 128–133, 10.15586/AEI.V53I4.1387. 40682237

[141] Ş. Hacioǧlu , E. B. Başkan , Ş. Tunali , and H. Saricaoǧlu , “Patch Test Results With Standard and Cosmetic Series in Patients With Suspected Cosmetic‐Induced Contact Dermatitis,” Turkderm Deri Hastaliklari Ve Frengi Arsivi 44, no. 4 (2010): 193–199, 10.4274/turkderm.44.193.

[142] Ö. Çalka , A. S. Karadağ , N. Akdeniz , and S. G. Bilgili , “The Results of Patch Testing in Patients With Contact Dermatitis in Eastern Turkey,” TURKDERM 45, no. 1 (2011): 19–23, 10.4274/turkderm.45.05.

[143] G. Erfan , M. E. Yanık , Ş. Kaya , S. Kalaycı , K. Taşolar , and M. Kulaç , “Patch Testing for Allergic Contact Dermatitis: Three Years Retrospective Results in Tekirdağ,” TURKDERM 49, no. 2 (2015): 129–133, 10.4274/turkderm.15689.

[144] S. Kundak , “Patch Test Results of Contact Sensitization in Children Without Atopic Dermatitis: A Single Tertiary Center Experience,” Dermatitis 31, no. 2 (2020): 153–156, 10.1097/DER.0000000000000530. 31609858

[145] Z. Y. Katran and İ. Bulut , “Results of Patch Testing to the European Baseline Series in Adult Patients in Turkey: A Five‐Year Experience at a Tertiary Reference Center,” Alergologia Polska ‐ Polish Journal of Allergology 11, no. 1 (2024): 10–16, 10.5114/pja.2024.135510.

[146] M. O. G. El‐Rab and O. A. Al‐Sheikh , “Is the European Standard Series Suitable for Patch Testing in Riyadh, Saudi Arabia?,” Contact Dermatitis 33, no. 5 (1995): 310–314, 10.1111/J.1600-0536.1995.TB02044.X. 8565485

[147] C. W. Lynde , L. Warshawski , and J. C. Mitchell , “Screening Patch Tests in 4190 Eczema Patients 1972–81,” Contact Dermatitis 8, no. 6 (1982): 417–421, 10.1111/j.1600-0536.1982.tb04271.x. 7172658

[148] D. J. Hogan , M. Hill , and P. R. Lane , “Results of Routine Patch Testing of 542 Patients in Saskatoon, Canada,” Contact Dermatitis 19, no. 2 (1988): 120–124, 10.1111/j.1600-0536.1988.tb05508.x. 3180777

[149] J. I. Silverberg , A. Hou , E. M. Warshaw , et al., “Age‐Related Differences in Patch Testing Results Among Children: Analysis of North American Contact Dermatitis Group Data, 2001‐2018,” Journal of the American Academy of Dermatology 86, no. 4 (2022): 818–826, 10.1016/j.jaad.2021.07.030. 34314743

[150] M. R. Albert , S. Gonzalez , and E. Gonzalez , “Patch Testing Reactions to a Standard Series in 608 Patients Tested From 1990 to 1997 at Massachusetts General Hospital,” American Journal of Contact Dermatitis 9, no. 4 (1998): 207–211, 10.1016/s1046-199x(98)90030-6. 9810020

[151] B. E. Anderson , T. C. Tan , and J. G. Marks , “Patch‐Test Reactions to Formaldehydes, Bioban, and Other Formaldehyde Releasers,” Dermatitis 18, no. 2 (2007): 92–95, 10.2310/6620.2007.06012. 17498414

[152] S. H. Nguyen , T. P. Dang , C. MacPherson , H. Maibach , and H. I. Maibach , “Prevalence of Patch Test Results From 1970 to 2002 in a Multi‐Centre Population in North America (NACDG),” Contact Dermatitis 58, no. 2 (2008): 101–106, 10.1111/j.1600-0536.2007.01281.x. 18186744

[153] A. Odhav and D. V. Belsito , “Is Quaternium‐15 a Formaldehyde Releaser? Correlation Between Positive Patch Test Reactions to Formaldehyde and Quaternium‐15,” Dermatitis 23, no. 1 (2012): 39–43, 10.1097/DER.0b013e31823d1785. 22653068

[154] D. F. Rodrigues , D. R. Neves , J. M. Pinto , M. F. F. Alves , and A. C. F. Fulgêncio , “Results of Patch‐Tests From Santa Casa de Belo Horizonte Dermatology Clinic, Belo Horizonte, Brazil, From 2003 to 2010,” Anais Brasileiros de Dermatologia 87, no. 5 (2012): 800–803, 10.1590/S0365-05962012000500028. 23044584

[155] I. A. G. Duarte , G. M. Tanaka , N. M. Suzuki , et al., “Patch Test Standard Series Recommended by the Brazilian Contact Dermatitis Study Group During the 2006‐2011 Period,” Anais Brasileiros de Dermatologia 88, no. 6 (2013): 1015–1018, 10.1590/ABD1806-4841.20132374. 24474122 PMC3900364

[156] S. Hirano and K. Yoshikawa , “Patch Testing With European and American Standard Allergens in Japanese Patients,” Contact Dermatitis 8, no. 1 (1982): 48–50, 10.1111/j.1600-0536.1982.tb04134.x. 7067439

[157] Y. Q. Liu , B. Zhao , L. H. Zhuang , and W. X. Fan , “Patch Test Reactions to the Chinese Standard Screening Allergens in 1,135 Patients Investigated for Allergic Contact Dermatitis,” American Journal of Contact Dermatitis 8, no. 3 (1997): 141–143, 10.1016/S1046-199X(97)90093-2. 9249281

[158] W. S. Lam , L. Y. Chan , S. C. K. Ho , L. Y. Chong , W. H. So , and T. W. Wong , “A Retrospective Study of 2585 Patients Patch Tested With the European Standard Series in Hong Kong (1995–99),” International Journal of Dermatology 47, no. 2 (2008): 128–133, 10.1111/J.1365-4632.2008.03437.X. 18211481

[159] P. H. Lin , Y. H. Tseng , and C. Y. Chu , “Changing Trends of Contact Allergens: A 40‐Year Retrospective Study From a Referral Centre in Northern Taiwan,” Contact Dermatitis 85, no. 1 (2021): 39–45, 10.1111/COD.13795. 33502013

[160] D. S. Shenoi , C. Srinivas , and C. Balachandran , “Results of Patch Testing With a Standard Series of Allergens at Manipal,” Indian Journal of Dermatology, Venereology and Leprology 60 (1994): 133.

[161] V. K. Sharma , A. Chakrabarti , and V. K. Sharma , “Common Contact Sensitizers in Chandigarh, India. A Study of 200 Patients With the European Standard Series,” Contact Dermatitis 38, no. 3 (1998): 127–131, 10.1111/j.1600-0536.1998.tb05677.x. 9536402

[162] S. Cheng , Y. H. Leow , C. L. Goh , and A. Goon , “Contact Sensitivity to Preservatives in Singapore: Frequency of Sensitization to 11 Common Preservatives 2006‐2011,” Dermatitis 25, no. 2 (2014): 77–82, 10.1097/DER.0000000000000031. 24603520

[163] H. M. Cheng , A. T. J. Goon , Y. H. Leow , T. Y. R. Chong , and S. W. N. Cheng , “Contact Sensitivity to Preservatives in Singapore: A Retrospective Study of Patch Test Data From 2012 to 2021,” Contact Dermatitis 91, no. 5 (2024): 445–447, 10.1111/COD.14648. 39031108

[164] E. T. Chow , A. M. Avolio , A. Lee , and R. Nixon , “Frequency of Positive Patch Test Reactions to Preservatives: The Australian Experience,” Australasian Journal of Dermatology 54, no. 1 (2013): 31–35, 10.1111/j.1440-0960.2012.00958.x. 23083503

[165] A. Mahfoudh , O. Elmaleel , H. Kalboussi , et al., “Influence of Age on Patch Tests Results,” Turkish Journal of Dermatology/Türk Dermatoloji Dergisi 11, no. 1 (2017): 12–16, 10.4274/tdd.3058.

[166] A. Pontén , K. Aalto‐Korte , T. Agner , et al., “Patch Testing With 2.0% (0.60 Mg/cm^2^) Formaldehyde Instead of 1.0% (0.30 Mg/cm^2^) Detects Significantly More Contact Allergy,” Contact Dermatitis 68, no. 1 (2013): 50–53, 10.1111/j.1600-0536.2012.02169.x. 23035891

[167] M. Isaksson , I. Ale , K. Andersen , et al., “Multicenter Patch Testing With a Resol Resin Based on Phenol and Formaldehyde Within the International Contact Dermatitis Research Group,” Dermatitis 26, no. 5 (2015): 230–234, 10.1097/DER.0000000000000137. 26367206

[168] M. Isaksson , I. Ale , K. E. Andersen , et al., “Patch Testing With Formaldehyde 2.0% (0.60 Mg/cm^2^) Detects More Contact Allergy to Formaldehyde Than 1.0%,” Dermatitis 30, no. 6 (2019): 342–346, 10.1097/DER.0000000000000510. 31730552

[169] D. Isufi , M. B. Jensen , C. Kursawe Larsen , F. Alinaghi , J. F. B. Schwensen , and J. D. Johansen , “Allergens Responsible for Contact Allergy in Children From 2010 to 2024: A Systematic Review and Meta‐Analysis,” Contact Dermatitis 92, no. 5 (2025): 327–343, 10.1111/COD.14753. 39827476 PMC11965549

[170] M. B. Jensen , D. Isufi , C. K. Larsen , J. F. B. Schwensen , F. Alinaghi , and J. D. Johansen , “Prevalence of Contact Allergy to Neomycin in Dermatitis Patients: A Systematic Review and Meta‐Analysis,” Contact Dermatitis 93, no. 1 (2025): 1–15, 10.1111/COD.14784. 40107276 PMC12134448

[171] J. L. Owen , P. P. Vakharia , and J. I. Silverberg , “The Role and Diagnosis of Allergic Contact Dermatitis in Patients With Atopic Dermatitis,” American Journal of Clinical Dermatology 19, no. 3 (2018): 293–302, 10.1007/S40257-017-0340-7. 29305764 PMC5948135

[172] C. R. Hamann , D. Hamann , A. Egeberg , J. D. Johansen , J. Silverberg , and J. P. Thyssen , “Association Between Atopic Dermatitis and Contact Sensitization: A Systematic Review and Meta‐Analysis,” Journal of the American Academy of Dermatology 77, no. 1 (2017): 70–78, 10.1016/j.jaad.2017.02.001. 28392290

[173] A. Goossens and O. Aerts , “Contact Allergy to and Allergic Contact Dermatitis From Formaldehyde and Formaldehyde Releasers: A Clinical Review and Update,” Contact Dermatitis 87, no. 1 (2022): 20–27, 10.1111/COD.14089. 35229319

[174] R. L. Rietschel , E. M. Warshaw , D. Sasseville , et al., “Sensitivity of Petrolatum and Aqueous Vehicles for Detecting Allergy to Imidazolidinylurea, Diazolidinylurea, and DMDM Hydantoin: A Retrospective Analysis From the North American Contact Dermatitis Group,” Dermatitis 18, no. 3 (2007): 155–162, 10.2310/6620.2007.06040. 17725923

[175] M. Kireche , J. L. Peiffer , D. Antonios , et al., “Evidence for Chemical and Cellular Reactivities of the Formaldehyde Releaser Bronopol, Independent of Formaldehyde Release,” Chemical Research in Toxicology 24, no. 12 (2011): 2115–2128, 10.1021/TX2002542. 22034943

[176] A. Trattner , J. D. Johansen , and T. Menné , “Formaldehyde Concentration in Diagnostic Patch Testing: Comparison of 1% With 2%,” Contact Dermatitis 38, no. 1 (1998): 9–13, 10.1111/J.1600-0536.1998.TB05630.X. 9504240

